# Fluticasone- vs Budesonide-Based Dual Therapy for COPD

**DOI:** 10.1001/jamanetworkopen.2026.0959

**Published:** 2026-03-09

**Authors:** William B. Feldman, Vidya L. Ambati, Samy Suissa, Aaron S. Kesselheim, Jerry Avorn, Sebastian Schneeweiss, Shirley V. Wang

**Affiliations:** 1 Division of Pharmacoepidemiology and Pharmacoeconomics, Department of Medicine, Brigham and Women’s Hospital, Boston, Massachusetts; 2 Division of Pulmonary and Critical Care Medicine, Department of Medicine, Brigham and Women’s Hospital, Boston, Massachusetts; 3 Harvard Medical School, Boston, Massachusetts; 4 Now with Division of Pulmonary, Critical Care, Sleep Medicine, Clinical Immunology, and Allergy, Department of Medicine, University of California, Los Angeles; 5 Centre for Clinical Epidemiology, Lady Davis Institute for Medical Research, Jewish General Hospital

## Abstract

**Importance:**

Inhaled corticosteroid (ICS)–long-acting β-agonist (LABA) inhalers are generally considered therapeutically equivalent when treating chronic obstructive pulmonary disease (COPD). However, metered-dose inhalers in the class are associated with substantially higher greenhouse gas emissions than dry powder formulations, and studies have raised questions about potential intraclass differences in clinical outcomes among patients receiving ICS-LABAs.

**Objective:**

To analyze COPD exacerbations and pneumonia hospitalizations associated with once-daily fluticasone furoate–vilanterol dry powder inhalers, twice-daily fluticasone propionate–salmeterol dry powder inhalers, and twice-daily budesonide-formoterol metered-dose inhalers in adults with COPD.

**Design, Setting, and Participants:**

This cohort study was conducted using longitudinal commercial claims data of US adults aged 40 years or older with COPD. Patients were 1:1 pairwise propensity score matched into 3 cohorts: (1) new users receiving fluticasone furoate–vilanterol vs budesonide-formoterol between January 1, 2014, and February 29, 2024; (2) new users receiving fluticasone furoate–vilanterol vs fluticasone propionate–salmeterol between January 1, 2014, and February 29, 2024; and (3) new users receiving fluticasone propionate–salmeterol vs budesonide-formoterol between January 1, 2007, and February 29, 2024.

**Exposures:**

Receipt of a once-daily fluticasone furoate–vilanterol dry powder inhaler (Breo Ellipta; GSK), twice-daily fluticasone propionate–salmeterol dry powder inhaler (Advair Diskus; GSK), or twice-daily budesonide-formoterol metered-dose inhaler (Symbicort; AstraZeneca).

**Main Outcomes and Measures:**

The primary outcomes were first moderate or severe COPD exacerbation and first pneumonia hospitalization. Hazard ratios and 95% CIs were estimated using Cox proportional hazards regression models.

**Results:**

The cohorts included 38 070 matched pairs of patients receiving fluticasone furoate–vilanterol vs budesonide-formoterol (58.8% women; mean [SD] age, 71.0 [9.0] years), 20 471 matched pairs of patients receiving fluticasone furoate–vilanterol vs fluticasone propionate–salmeterol (58.3% women; mean [SD] age, 69.9 [9.2] years), and 55 627 matched pairs of patients receiving fluticasone propionate–salmeterol vs budesonide-formoterol (56.2% women; mean [SD] age, 68.3 [9.0] years). Patients receiving fluticasone furoate–vilanterol had a 9% lower risk of moderate or severe COPD exacerbations compared with those receiving budesonide-formoterol (hazard ratio [HR], 0.91 [95% CI, 0.88-0.94]; number needed to treat [NNT] = 40) and a 6% lower risk compared with those receiving fluticasone propionate–salmeterol (HR, 0.94 [95% CI, 0.89-0.98]; NNT = 40). The risk of moderate or severe COPD exacerbation was similar for patients receiving fluticasone propionate–salmeterol and budesonide-formoterol (HR, 0.98 [95% CI, 0.95-1.01]). No differences were observed in the risk of pneumonia hospitalization across the 3 cohorts (fluticasone furoate–vilanterol vs budesonide-formoterol: HR, 1.03 [95% CI, 0.96-1.11]; fluticasone furoate–vilanterol vs fluticasone propionate–salmeterol: HR, 0.93 [95% CI, 0.85-1.03]; and fluticasone propionate–salmeterol vs budesonide-formoterol: HR, 1.04 [95% CI, 0.98-1.10]).

**Conclusions and Relevance:**

In this cohort study of new ICS-LABA users with COPD, once-daily dry powder fluticasone furoate–vilanterol inhalers were associated with slightly improved clinical outcomes compared with twice-daily metered-dose budesonide-formoterol inhalers and twice-daily dry powder fluticasone propionate–salmeterol inhalers. Further studies are needed to explore potential intraclass differences among inhalers used to treat COPD.

## Key Points

**Question:**

Are different inhaled corticosteroid (ICS)–long-acting β-agonist (LABA) inhalers associated with different clinical outcomes among patients with chronic obstructive pulmonary disease (COPD)?

**Findings:**

In this cohort study of new ICS-LABA users, the dry powder inhaler fluticasone furoate–vilanterol was associated with a 9% lower risk of first moderate or severe COPD exacerbation compared with the metered-dose inhaler budesonide-formoterol and a 6% lower risk compared with the dry powder inhaler fluticasone propionate–salmeterol. Fluticasone propionate–salmeterol and budesonide-formoterol were associated with clinical outcomes that were similar to one another.

**Meaning:**

These findings suggest that dry powder fluticasone-containing ICS-LABAs, which generate less than one-twentieth the greenhouse gas emissions of metered-dose formulations, have similar or slightly improved clinical outcomes compared with budesonide-formoterol among new users with COPD.

## Teaser

This cohort study compares chronic obstructive pulmonary disease (COPD) exacerbations and pneumonia hospitalizations among adults receiving fluticasone furoate–vilanterol dry powder inhalers, fluticasone propionate–salmeterol dry powder inhalers, and budesonide-formoterol metered-dose inhalers for COPD.

## Introduction

Several combined inhaled corticosteroid (ICS)–long-acting β-agonists (LABAs) are approved to treat chronic obstructive pulmonary disease (COPD). These inhalers are available in both dry powder and metered-dose formulations. However, because the former are associated with less than one-twentieth the greenhouse gas emissions of the latter, some health systems have sought to increase prescribing of dry powder inhalers compared with metered-dose alternatives.^1,2,3,4^ Consensus international guidelines generally consider inhalers in the same class to be therapeutically equivalent in patients with COPD,^5^ but questions persist about the comparative effectiveness and safety of dry powder vs metered-dose ICS-LABAs given the lack of head-to-head trials.

Previous cohort studies have suggested that fluticasone-based ICS-LABAs may be associated with reduced effectiveness^6,7,8,9,10,11^ and increased pneumonia risk^6,7,12,13,14,15,16,17^ compared with budesonide-based products. However, many earlier observational studies either did not control for device type or focused on delivery devices that are not available in the US, leaving open questions about heterogeneous treatment effects related to modes of drug delivery among inhalers on the US market. In addition, most prior research analyzed only fluticasone propionate, a short-acting medication that requires twice-daily dosing, rather than fluticasone furoate, a longer-acting version that requires just once-daily dosing. In contrast to studies analyzing fluticasone propionate–based therapies, researchers in one small 2019 retrospective study found that patients receiving fluticasone furoate–vilanterol had slightly improved clinical outcomes compared with those receiving budesonide-formoterol.^18^ However, the authors adjusted for a limited set of covariates, raising concerns for potential bias, and ended follow-up more than a decade ago without performing direct comparisons of fluticasone furoate–vilanterol and fluticasone propionate–salmeterol. In a 2024 study of fluticasone furoate–based single-inhaler triple therapy, researchers found a slightly reduced risk of moderate or severe COPD exacerbations compared with budesonide-based triple therapy and an identical risk of pneumonia hospitalization.^19^ However, that study analyzed inhalers with a long-acting muscarinic antagonist (LAMA) in addition to an ICS and LABA, thus making it difficult to draw conclusions about ICS-LABAs.

Given ongoing clinical uncertainty among fluticasone- and budesonide-based ICS-LABAs in the treatment of COPD, we analyzed the clinical outcomes associated with the 3 most commonly used products in the US: the once-daily dry powder inhaler fluticasone furoate–vilanterol (Breo Ellipta; GSK), the twice-daily dry powder inhaler fluticasone propionate–salmeterol (Advair Diskus; GSK), and the twice-daily metered-dose inhaler budesonide-formoterol (Symbicort; AstraZeneca).

## Methods

This cohort study was approved by the Mass General Brigham Institutional Review Board. Informed consent was waived because the analysis used deidentified data. The protocol was preregistered with the Center for Open Science (https://osf.io/yp2kh) before the analyses were implemented. The study followed the Strengthening the Reporting of Observational Studies in Epidemiology (STROBE) reporting guideline.

### Study Cohort

We performed new-user cohort studies using data from a large insurance claims database (Optum Clinformatics Data Mart), which includes data from more than 90 million patients across the US with commercial and Medicare Advantage health plans. To analyze the 3 different ICS-LABA inhalers selected for investigation, we generated 3 distinct cohorts of patients: (1) a cohort of new users receiving fluticasone furoate–vilanterol (exposure) vs budesonide-formoterol (referent) between January 1, 2014, and February 29, 2024; (2) a cohort of new users receiving fluticasone furoate–vilanterol (exposure) vs fluticasone propionate–salmeterol (referent) between January 1, 2014, and February 29, 2024; and (3) a cohort of new users receiving fluticasone propionate–salmeterol (exposure) vs budesonide-formoterol (referent) between January 1, 2007, and February 29, 2024. We included only patients initiating the moderate strength version of each inhaler, as this is the only strength approved for the treatment of COPD (eMethods in Supplement 1). To identify new users of ICS-LABA therapy, we excluded patients who had filled an ICS-LABA prescription in the 365 days before cohort entry and those who had received another maintenance inhaler besides the exposure or reference product on the cohort entry date.

The complete inclusion and exclusion criteria, including definitions based on *International Classification of Diseases, Ninth Revision, Clinical Modification* (*ICD-9-CM*) and *International Classification of Diseases, Tenth Revision, Clinical Modification* (*ICD-10-CM*) diagnosis codes, are provided in eTable 1 in Supplement 1. To enter the cohort, all patients must have been enrolled in an insurance plan in the dataset for at least 365 days, had an active diagnosis of COPD, and been at least 40 years of age.

### Assessment of Covariates

We measured potential confounders during the 365 days leading up to and including the cohort entry date. These potential confounders included indicators of baseline lung disease, comorbidities, general health care use, and receipt of other medications (the eMethods in Supplement 1 provides a complete list of covariates included in the propensity score model). We measured prior asthma diagnosis codes using all available data and eosinophil levels based on the last observed value in the 180 days before cohort entry. Covariates for age, sex, region, calendar year, season, and prescriber specialty were measured on the cohort entry date. A visualization of the temporality of covariate assessment windows is provided in eFigure 1 in Supplement 1.

### Primary Outcomes and Follow-Up

The primary effectiveness outcome was a first moderate or severe COPD exacerbation, and the primary safety outcome was a first hospitalization for pneumonia. Following Global Obstructive Lung Disease (GOLD) guidelines^5^ and numerous randomized clinical trials and observational studies,^20,21,22,23,24,25,26,27^ moderate exacerbations were episodes requiring systemic glucocorticoids, while severe exacerbations were episodes requiring hospitalization for COPD. Moderate exacerbations were measured based on 5- to 14-day fills of an oral glucocorticoid (prednisone, prednisolone, or methylprednisolone) (positive predictive value [PPV] = 0.73),^28^ and severe exacerbations were based on *ICD-9-CM* (491.xx, 492.xx, or 496) and *ICD-10-CM* (J41.x, J42.x, J43.x, or J44.x) codes in the primary diagnosis position for the hospitalization (PPV = 0.86) (eMethods in Supplement 1).^29^ Pneumonia hospitalizations were defined using *ICD-9-CM* (480.x-488.xx) and *ICD-10-CM* (J.09.X1, J10.xx-J18.x, A01.03, A02.22, A37.01, A37.11, A37.81, A37.91, A54.84, B01.2, B05.2, B06.81, B77.81, J85.1, or J22) codes in any position (PPV = 0.88).^30^ The study was designed as an on-treatment analysis in which we followed up patients for up to 1 year after cohort entry. The causal estimand of interest was per protocol; hence, patients were censored for any of the following events: experiencing the outcome of interest, treatment discontinuation with a grace period of 60 days permitted between inhaler fills and an exposure risk window of 60 days, the addition or switch to a new maintenance inhaler, death, or insurance disenrollment.

### Prespecified Secondary, Subgroup, and Sensitivity Analysis

We performed several analyses of secondary end points, including (1) first moderate exacerbation, (2) first severe exacerbation, (3) all-cause mortality, (4) annual rate of moderate or severe exacerbations, and (5) annual rate of pneumonia hospitalizations. We also performed subgroup analyses based on measures of disease severity, eosinophil levels, co-occurring asthma, receipt of spirometry, and whether the index prescription had been written by a pulmonologist (eMethods in Supplement 1).

We tested the robustness of our findings by conducting several prespecified sensitivity analyses, including varying the grace period permitted between prescription fills (from 60 days to 30 days and 90 days), applying an as-started design in which patients were followed for up to 1 year regardless of whether they discontinued treatment or switched therapies, excluding early events (in the first 30 days and first 60 days after cohort entry), limiting follow-up to 180 days, varying the outcome definitions of moderate exacerbations in 3 different ways (requiring a clinic or emergency department visit before the oral steroid prescription, requiring both a steroid and an antibiotic prescription, and permitting either a steroid or an antibiotic prescription), varying the outcome definition of severe exacerbations (to require an exacerbation code in any position of the hospitalization), and varying the outcome definition of pneumonia hospitalizations (to require a diagnosis code for pneumonia in the primary position). We performed post hoc sensitivity analyses that included all strengths of each inhaler (not just the strengths approved for COPD) and adjusted for these strengths in the propensity score model.

### Statistical Analysis

We estimated a propensity score for each comparison by fitting a logistic regression model including all preexposure variables without further selection, and we performed 1:1 propensity score matching using logistic regression and nearest-neighbor matching with a caliper of 0.01 on the propensity score scale. We applied a missing indicator whenever data were missing for any covariates in the model. We estimated hazard ratios (HRs) and 95% CIs using a Cox proportional hazards regression model in our time-to-event analyses. Both unadjusted and propensity score matched analyses are presented. We used Kaplan-Meier curves and plots of Schoenfeld residuals to visually inspect whether hazards were proportional. The number needed to treat (NNT) was calculated as 1/absolute risk difference at 365 days, where the absolute risks in the treatment arms were estimated from Kaplan-Meier curves.^31^ When comparing the annual rates of COPD exacerbations and pneumonia hospitalizations, we performed negative binomial regression. We also conducted prespecified sensitivity analyses using 1:1 high-dimensional propensity score matching, which can help reduce bias from unmeasured confounders by relying on an automated selection procedure to adjust for hundreds of covariates in addition to prespecified measures derived from outpatient and inpatient diagnosis codes, procedures, and pharmacy claims.^32,33,34^

All analyses were completed using the Aetion Evidence Platform, version 5 (Aetion Inc), which has been validated in prior studies and trial emulations,^35,36^ and Stata, version 16 (StataCorp LLC).

## Results

The cohorts included 38 070 matched pairs of patients receiving fluticasone furoate–vilanterol vs budesonide-formoterol (58.8% women and 41.2% men; mean [SD] age, 71.0 [9.0] years), 20 471 matched pairs of patients receiving fluticasone furoate–vilanterol vs fluticasone propionate–salmeterol (58.3% women and 41.7% men; mean [SD] age, 69.9 [9.2] years), and 55 627 matched pairs of patients receiving fluticasone propionate–salmeterol vs budesonide-formoterol (56.2% women and 43.8% men; mean [SD] age, 68.3 [9.0] years) (Table 1 and eFigure 2 in Supplement 1). The cohorts were already well balanced before propensity score matching (eTable 2 in Supplement 1), with mean absolute standardized differences among covariates of 0.035, 0.089, and 0.095, respectively. After propensity score matching, mean absolute standardized differences among covariates were 0.003, 0.005, and 0.004 (eFigure 3 in Supplement 1 provides the propensity score distributions).

**Table 1.** Baseline Characteristics of Patients in the Matched Cohorts Initiating ICS-LABA Therapy^a^

Characteristic	Cohort								
	FF-V vs B-F (2014-2024; n = 38 070 matched pairs)			FF-V vs FP-S (2014-2024; n = 20 471 matched pairs)			FP-S vs B-F (2007-2024; n = 55 627 matched pairs)		
	B-F	FF-V	Absolute standardized difference	FP-S	FF-V	Absolute standardized difference	B-F	FP-S	Absolute standardized difference
Age, mean (SD), y	71.0 (8.9)	71.0 (9.2)	0.002	70.0 (9.1)	69.9 (9.3)	0.012	68.3 (9.0)	68.4 (8.9)	0.018
Sex									
Female	22 450 (59.0)	22 311 (58.6)	0.007	11 978 (58.5)	11 900 (58.1)	0.008	31 219 (56.1)	31 323 (56.3)	0.004
Male	15 617 (41.0)	15 757 (41.4)	0.007	8491 (41.5)	8569 (41.9)	0.008	24 395 (43.9)	24 291 (43.7)	0.004
Missing	3 (<0.001)	2 (<0.001)	0.003	2 (<0.001)	2 (<0.001)	<0.001	13 (<0.001)	13 (<0.001)	<0.001
US region									
Northeast	4566 (12.0)	4600 (12.1)	0.003	2290 (11.2)	2317 (11.3)	0.004	5606 (10.1)	5711 (10.3)	0.006
Midwest	7696 (20.2)	7597 (20.0)	0.006	3987 (19.5)	3953 (19.3)	0.004	10 900 (19.6)	10 830 (19.5)	0.003
South	19 130 (50.2)	19 214 (50.5)	0.004	9813 (47.9)	9841 (48.1)	0.003	25 916 (46.6)	25 749 (46.3)	0.006
West	6600 (17.3)	6584 (17.3)	0.001	4337 (21.2)	4300 (21.0)	0.004	13 065 (23.5)	13 210 (23.7)	0.006
Missing	78 (0.2)	75 (0.2)	0.002	44 (0.2)	60 (0.3)	0.016	140 (0.3)	127 (0.2)	0.005
Season of cohort entry									
Winter	9960 (26.2)	9883 (26.0)	0.005	5353 (26.1)	5441 (26.6)	0.010	14 751 (26.5)	14 667 (26.4)	0.003
Spring	10 182 (26.7)	10 216 (26.8)	0.002	5562 (27.2)	5485 (26.8)	0.008	14 876 (26.7)	14 756 (26.5)	0.005
Summer	9038 (23.7)	9099 (23.9)	0.004	4793 (23.4)	4778 (23.3)	0.002	12 954 (23.3)	13 026 (23.4)	0.003
Fall	8890 (23.4)	8872 (23.3)	0.001	4763 (23.3)	4767 (23.3)	<0.001	13 046 (23.5)	13 178 (23.7)	0.006
Year of cohort entry									
2007	NA	NA	NA	NA	NA	NA	309 (0.6)	239 (0.4)	0.018
2008	NA	NA	NA	NA	NA	NA	1244 (2.2)	1055 (1.9)	0.024
2009	NA	NA	NA	NA	NA	NA	2996 (5.4)	2960 (5.3)	0.003
2010	NA	NA	NA	NA	NA	NA	4046 (7.3)	4054 (7.3)	0.001
2011	NA	NA	NA	NA	NA	NA	4103 (7.4)	4197 (7.5)	0.006
2012	NA	NA	NA	NA	NA	NA	4760 (8.6)	4883 (8.8)	0.008
2013	NA	NA	NA	NA	NA	NA	5609 (10.1)	5755 (10.3)	0.009
2014	1282 (3.4)	1279 (3.4)	<0.001	1197 (5.8)	1277 (6.2)	0.016	5325 (9.6)	5389 (9.7)	0.004
2015	2406 (6.3)	2375 (6.2)	0.003	2303 (11.3)	2341 (11.4)	0.006	4996 (9.0)	5067 (9.1)	0.004
2016	3313 (8.7)	3363 (8.8)	0.005	3215 (15.7)	3137 (15.3)	0.011	5509 (9.9)	5471 (9.8)	0.002
2017	4906 (12.9)	4844 (12.7)	0.005	4325 (21.1)	4268 (20.8)	0.007	6132 (11.0)	6091 (10.9)	0.002
2018	5187 (13.6)	5226 (13.7)	0.003	4270 (20.9)	4308 (21.0)	0.005	5529 (9.9)	5434 (9.8)	0.006
2019	5016 (13.2)	5023 (13.2)	0.001	1770 (8.6)	1786 (8.7)	0.003	1660 (3.0)	1718 (3.1)	0.006
2020	4247 (11.2)	4167 (10.9)	0.007	817 (4.0)	825 (4.0)	0.002	818 (1.5)	792 (1.4)	0.004
2021	4325 (11.4)	4329 (11.4)	<0.001	858 (4.2)	821 (4.0)	0.009	862 (1.5)	847 (1.5)	0.002
2022	3571 (9.4)	3678 (9.7)	0.010	853 (4.2)	845 (4.1)	0.002	818 (1.5)	824 (1.5)	0.001
2023	3252 (8.5)	3235 (8.5)	0.002	818 (4.0)	806 (3.9)	0.003	857 (1.5)	807 (1.5)	0.007
2024	565 (1.5)	551 (1.4)	0.003	45 (0.2)	57 (0.3)	0.012	54 (0.1)	44 (0.1)	0.006
Baseline lung disease									
GOLD Group E disease	8531 (22.4)	8437 (22.2)	0.006	4718 (23.0)	4730 (23.1)	0.001	12 929 (23.2)	12 891 (23.2)	0.002
COPD exacerbations, mean (SD)									
Moderate	0.71 (1.07)	0.70 (1.05)	0.009	0.70 (1.07)	0.70 (1.04)	0.005	0.68 (1.03)	0.67 (1.05)	0.004
Severe	0.08 (0.32)	0.08 (0.33)	0.001	0.09 (0.34)	0.09 (0.34)	0.001	0.10 (0.36)	0.10 (0.37)	0.001
Fills, mean (SD)									
SABA	2.03 (3.19)	2.02 (3.25)	0.004	2.09 (3.35)	2.11 (3.47)	0.007	2.14 (3.52)	2.13 (3.54)	0.004
SAMA	0.08 (0.64)	0.08 (0.67)	0.004	0.09 (0.72)	0.09 (0.71)	0.001	0.14 (0.97)	0.14 (0.99)	0.001
SAMA-SABA	0.55 (1.88)	0.54 (1.94)	0.006	0.61 (2.14)	0.60 (2.03)	0.004	0.71 (2.27)	0.72 (2.37)	0.004
Pneumonia hospitalizations, mean (SD)	0.14 (0.48)	0.14 (0.48)	0.002	0.14 (0.49)	0.14 (0.47)	0.006	0.13 (0.46)	0.13 (0.45)	0.003
Respiratory antibiotic fills, mean (SD)	1.70 (2.02)	1.68 (2.00)	0.006	1.73 (2.04)	1.74 (1.99)	0.003	1.87 (2.10)	1.86 (2.13)	0.002
Any prior claim for asthma^b^	16 919 (44.4)	16 787 (44.1)	0.007	9415 (46.0)	9445 (46.1)	0.003	26 891 (48.3)	26 911 (48.4)	0.001
Home oxygen or equipment claim	8172 (21.5)	8125 (21.3)	0.003	4493 (21.9)	4494 (22.0)	<0.001	13 282 (23.9)	13 223 (23.8)	0.002
CPAP or BiPAP	4049 (10.6)	3990 (10.5)	0.005	1828 (8.9)	1864 (9.1)	0.006	4565 (8.2)	4528 (8.1)	0.002
Spirometry	14 363 (37.7)	14 254 (37.4)	0.006	6858 (33.5)	7037 (34.4)	0.018	18 969 (34.1)	18 843 (33.9)	0.005
Index prescription by pulmonologist	3565 (9.4)	3483 (9.1)	0.007	1221 (6.0)	1258 (6.1)	0.008	3142 (5.6)	3168 (5.7)	0.002
Smoking	22 392 (58.8)	22 352 (58.7)	0.002	11 528 (56.3)	11 530 (56.3)	<0.001	26 128 (47.0)	26 087 (46.9)	0.001
Pulmonary rehabilitation	264 (0.7)	264 (0.7)	<0.001	123 (0.6)	133 (0.6)	0.006	338 (0.6)	348 (0.6)	0.002
Inhaler use									
LAMA	5579 (14.7)	5538 (14.5)	0.003	3492 (17.1)	3514 (17.2)	0.003	10 805 (19.4)	10 832 (19.5)	0.001
LABA	446 (1.2)	444 (1.2)	<0.001	285 (1.4)	276 (1.3)	0.004	1200 (2.2)	1183 (2.1)	0.002
ICS	2805 (7.4)	2806 (7.4)	<0.001	1493 (7.3)	1512 (7.4)	0.004	5111 (9.2)	5045 (9.1)	0.004
LAMA-LABA	1849 (4.9)	1849 (4.9)	<0.001	576 (2.8)	611 (3.0)	0.010	676 (1.2)	587 (1.1)	0.015
ICS-LAMA-LABA	1083 (2.8)	1089 (2.9)	0.001	187 (0.9)	207 (1.0)	0.010	190 (0.3)	178 (0.3)	0.004
Any maintenance inhaler	10 153 (26.7)	10 127 (26.6)	0.002	5265 (25.7)	5321 (26.0)	0.006	15 454 (27.8)	15 365 (27.6)	0.004
Chronic azithromycin	254 (0.7)	239 (0.6)	0.005	113 (0.6)	119 (0.6)	0.004	301 (0.5)	286 (0.5)	0.004
Roflumilast	173 (0.5)	154 (0.4)	0.008	79 (0.4)	86 (0.4)	0.005	197 (0.4)	192 (0.3)	0.002
Chronic oral steroids	2527 (6.6)	2534 (6.7)	0.001	1393 (6.8)	1367 (6.7)	0.005	4393 (7.9)	4416 (7.9)	0.002
Events within 30 d of cohort entry									
Moderate or severe COPD exacerbation	6891 (18.1)	6906 (18.1)	0.001	4130 (20.2)	4146 (20.3)	0.002	12 011 (21.6)	11 969 (21.5)	0.002
Respiratory antibiotic fill	8804 (23.1)	8733 (22.9)	0.004	5182 (25.3)	5146 (25.1)	0.004	16 163 (29.1)	16 108 (29.0)	0.002
Baseline eosinophil count									
CBC with differential performed	24 229 (63.6)	24 202 (63.6)	0.001	12 353 (60.3)	12 324 (60.2)	0.003	30 719 (55.2)	30 860 (55.5)	0.005
Eosinophil categories, No./μL									
≤100	3380 (8.9)	3442 (9.0)	0.006	1816 (8.9)	1841 (9.0)	0.004	3874 (7.0)	3950 (7.1)	0.005
>100 and ≤300	5010 (13.2)	4943 (13.0)	0.005	2606 (12.7)	2538 (12.4)	0.010	5159 (9.3)	5183 (9.3)	0.001
>300	2103 (5.5)	2119 (5.6)	0.002	1084 (5.3)	1077 (5.3)	0.002	2190 (3.9)	2221 (4.0)	0.003
Missing	27 577 (72.4)	27 566 (72.4)	0.001	14 965 (73.1)	15 015 (73.3)	0.006	44 404 (79.8)	44 273 (79.6)	0.006
Other comorbidities									
Combined comorbidity score, mean (SD)^c^	4.28 (3.38)	4.26 (3.40)	0.004	4.01 (3.28)	3.99 (3.32)	0.007	3.41 (3.07)	3.43 (3.05)	0.006
Frailty score, mean (SD)^d^	0.21 (0.07)	0.21 (0.08)	0.002	0.21 (0.07)	0.21 (0.07)	0.002	0.21 (0.07)	0.21 (0.07)	0.004
Obstructive sleep apnea	8541 (22.4)	8489 (22.3)	0.003	3892 (19.0)	3987 (19.5)	0.012	8677 (15.6)	8654 (15.6)	0.001
Hypertension	31 205 (82.0)	31 316 (82.3)	0.008	16 524 (80.7)	16 490 (80.6)	0.004	43 669 (78.5)	43 675 (78.5)	<0.001
Diabetes	13 945 (36.6)	13 993 (36.8)	0.003	7434 (36.3)	7431 (36.3)	<0.001	19 292 (34.7)	19 314 (34.7)	0.001
Obesity	10 312 (27.1)	10 324 (27.1)	0.001	5011 (24.5)	5069 (24.8)	0.007	10 285 (18.5)	10 298 (18.5)	0.001
Coronary artery disease	14 541 (38.2)	14 525 (38.2)	0.001	7287 (35.6)	7301 (35.7)	0.001	19 058 (34.3)	19 048 (34.2)	<0.001
Peripheral vascular disease	12 002 (31.5)	11 968 (31.4)	0.002	6106 (29.8)	6074 (29.7)	0.003	14 057 (25.3)	14 162 (25.5)	0.004
Venous thromboembolic disease	1738 (4.6)	1771 (4.7)	0.004	946 (4.6)	946 (4.6)	<0.001	2760 (5.0)	2765 (5.0)	<0.001
Congestive heart failure	11 245 (29.5)	11 238 (29.5)	<0.001	5714 (27.9)	5691 (27.8)	0.003	14 924 (26.8)	15 075 (27.1)	0.006
Gastroesophageal reflux disease	13 936 (36.6)	13 891 (36.5)	0.002	6876 (33.6)	6923 (33.8)	0.005	16 871 (30.3)	16 898 (30.4)	0.001
Kidney failure	9246 (24.3)	9194 (24.2)	0.003	4905 (24.0)	4874 (23.8)	0.004	11 058 (19.9)	11 133 (20.0)	0.003
Osteoporosis	4139 (10.9)	4088 (10.7)	0.004	2018 (9.9)	2004 (9.8)	0.002	4982 (9.0)	5070 (9.1)	0.006
Dementia or other neurologic disease	4036 (10.6)	4006 (10.5)	0.003	1937 (9.5)	1889 (9.2)	0.008	3118 (5.6)	3117 (5.6)	<0.001
Malignant neoplasm, nonmetastatic	5464 (14.4)	5484 (14.4)	0.001	2808 (13.7)	2806 (13.7)	<0.001	7651 (13.8)	7700 (13.8)	0.003
Metastatic solid organ malignant neoplasm	1021 (2.7)	1006 (2.6)	0.002	502 (2.5)	494 (2.4)	0.003	1321 (2.4)	1307 (2.3)	0.002
Anxiety disorder	10 728 (28.2)	10 721 (28.2)	<0.001	5395 (26.4)	5344 (26.1)	0.006	11 770 (21.2)	11 820 (21.2)	0.002
Depression	10 761 (28.3)	10 664 (28.0)	0.006	5569 (27.2)	5531 (27.0)	0.004	12 091 (21.7)	12 233 (22.0)	0.006
Health care utilization									
Emergency department visits, mean (SD)	2.73 (4.61)	2.71 (4.64)	0.003	2.69 (4.46)	2.68 (4.79)	0.002	2.57 (4.92)	2.60 (5.08)	0.005
Hospitalizations, mean (SD)	0.78 (1.47)	0.78 (1.52)	0.002	0.77 (1.47)	0.77 (1.52)	<0.001	0.74 (1.45)	0.74 (1.40)	<0.001
90-d Readmissions, mean (SD)	0.18 (0.72)	0.18 (0.75)	0.001	0.18 (0.71)	0.17 (0.73)	0.002	0.18 (0.76)	0.18 (0.74)	<0.001
Office visits, mean (SD)	12.34 (9.18)	12.31 (9.07)	0.003	11.76 (9.15)	11.78 (8.73)	0.003	11.50 (8.69)	11.57 (8.98)	0.008
Pulmonology visits, mean (SD)	0.28 (0.97)	0.28 (0.96)	0.010	0.21 (0.81)	0.22 (0.87)	0.012	0.20 (0.85)	0.20 (0.87)	0.001
Prescription claims, mean (SD)	52.30 (39.69)	52.35 (41.75)	0.001	52.70 (41.67)	52.92 (42.89)	0.005	52.36 (39.36)	52.48 (39.98)	0.003
Basic or complete metabolic panel	33 339 (87.6)	33 333 (87.6)	<0.001	17 392 (85.0)	17 369 (84.8)	0.003	44 752 (80.5)	44 846 (80.6)	0.004
Electrocardiogram	25 225 (66.3)	25 276 (66.4)	0.003	13 248 (64.7)	13 322 (65.1)	0.008	35 427 (63.7)	35 562 (63.9)	0.005
Echocardiogram	14 490 (38.1)	14 494 (38.1)	<0.001	7137 (34.9)	7162 (35.0)	0.003	18 765 (33.7)	18 823 (33.8)	0.002
CT scan	19 200 (50.4)	19 209 (50.5)	<0.001	9547 (46.6)	9597 (46.9)	0.005	24 377 (43.8)	24 483 (44.0)	0.004
Bronchoscopy or biopsy	1064 (2.8)	1044 (2.7)	0.003	502 (2.5)	519 (2.5)	0.005	1614 (2.9)	1568 (2.8)	0.005
Mammography	8079 (21.2)	8071 (21.2)	0.001	4288 (20.9)	4308 (21.0)	0.002	11 483 (20.6)	11 461 (20.6)	0.001
Colonoscopy	268 (0.7)	273 (0.7)	0.002	161 (0.8)	161 (0.8)	<0.001	579 (1.0)	574 (1.0)	0.001
Bone-mineral density scan	3662 (9.6)	3700 (9.7)	0.003	1875 (9.2)	1862 (9.1)	0.002	4921 (8.8)	4895 (8.8)	0.002
Influenza vaccination	22 198 (58.3)	22 223 (58.4)	0.001	11 655 (56.9)	11 530 (56.3)	0.012	27 864 (50.1)	28 066 (50.5)	0.007
Nonpulmonary medication									
Statins	23 004 (60.4)	23 032 (60.5)	0.002	11 816 (57.7)	11 844 (57.9)	0.003	29 812 (53.6)	29 938 (53.8)	0.005
β-Blockers	17 283 (45.4)	17 342 (45.6)	0.003	8794 (43.0)	8846 (43.2)	0.005	22 229 (40.0)	22 299 (40.1)	0.003
Angiotensin-converting enzyme inhibitors	10 877 (28.6)	10 858 (28.5)	0.001	6277 (30.7)	6264 (30.6)	0.001	18 583 (33.4)	18 618 (33.5)	0.001
Angiotensin receptor blockers	10 017 (26.3)	9963 (26.2)	0.003	4863 (23.8)	4828 (23.6)	0.004	11 370 (20.4)	11 393 (20.5)	0.001
Calcium channel blockers	12 420 (32.6)	12 403 (32.6)	0.001	6342 (31.0)	6361 (31.1)	0.002	16 552 (29.8)	16 537 (29.7)	0.001
Thiazide diuretics	8909 (23.4)	8926 (23.4)	0.001	4845 (23.7)	4819 (23.5)	0.003	13 940 (25.1)	13 973 (25.1)	0.001
Loop diuretics	11 050 (29.0)	11 097 (29.1)	0.003	5812 (28.4)	5728 (28.0)	0.009	14 982 (26.9)	15 085 (27.1)	0.004
Proton-pump inhibitors	15 527 (40.8)	15 476 (40.7)	0.003	7975 (39.0)	8006 (39.1)	0.003	20 790 (37.4)	20 918 (37.6)	0.005
H_2_-receptor blockers	3608 (9.5)	3560 (9.4)	0.004	1783 (8.7)	1769 (8.6)	0.002	4078 (7.3)	4109 (7.4)	0.002
Metformin	6348 (16.7)	6375 (16.7)	0.002	3426 (16.7)	3371 (16.5)	0.007	8603 (15.5)	8563 (15.4)	0.002
Sulfonylureas	2910 (7.6)	2893 (7.6)	0.002	1649 (8.1)	1631 (8.0)	0.003	4842 (8.7)	4794 (8.6)	0.003
Sodium-glucose cotransporter-2 inhibitors	899 (2.4)	902 (2.4)	0.001	290 (1.4)	308 (1.5)	0.007	416 (0.7)	377 (0.7)	0.008
Dipeptidyl peptidase-4 inhibitors	1587 (4.2)	1566 (4.1)	0.003	810 (4.0)	823 (4.0)	0.003	1905 (3.4)	1892 (3.4)	0.001
Glucagon-like peptide-1 agonists	1170 (3.1)	1212 (3.2)	0.006	468 (2.3)	452 (2.2)	0.005	793 (1.4)	768 (1.4)	0.004
Benzodiazepines	9159 (24.1)	9121 (24.0)	0.002	5244 (25.6)	5191 (25.4)	0.006	12 257 (22.0)	12 320 (22.1)	0.003
SSRIs or SNRIs	13 205 (34.7)	13 175 (34.6)	0.002	6902 (33.7)	6805 (33.2)	0.010	17 374 (31.2)	17 539 (31.5)	0.006
Socioeconomic covariate^e^									
Copayment on other medications, mean (SD), US $	20.75 (32.10)	20.72 (33.07)	0.001	20.99 (29.68)	20.98 (27.25)	0.001	23.35 (28.11)	23.25 (27.61)	0.004
Total copayment on other medications, mean (SD), US $	555.84 (871.81)	558.23 (930.06)	0.003	553.82 (785.51)	554.49 (769.81)	0.001	621.79 (800.04)	621.67 (856.96)	<0.001
Ratio of proprietary-to-generic medication	0.19 (0.25)	0.19 (0.24)	0.003	0.20 (0.28)	0.21 (0.25)	0.004	0.31 (0.43)	0.31 (0.44)	0.002

Abbreviations: B-F, budesonide-formoterol; BiPAP, bilevel positive airway pressure; CBC, complete blood count; COPD, chronic obstructive pulmonary disease; CPAP, continuous pressure airway pressure; CT, computed tomography; FF-V, fluticasone furoate–vilanterol; FP-S, fluticasone propionate–salmeterol; GOLD, Global Obstructive Lung Disease; ICS, inhaled corticosteroid; LABA, long-acting β-agonist; LAMA, long-acting muscarinic antagonist; NA, not applicable; SABA, short-acting β-agonist; SAMA, short-acting muscarinic antagonist; SNRI, serotonin and norepinephrine reuptake inhibitor; SSRI, selective serotonin reuptake inhibitor.

^a^ Unless otherwise indicated, values are presented as No. (%) of patients.

^b^ This covariate was measured using all available data for each patient (eFigure 1 in Supplement 1).

^c^ Combined comorbidity scores were calculated according to Gagne et al.^37^

^d^ Frailty index scores were calculated according to Kim et al.^38^

^e^ These covariates exclude out-of-pocket costs on the day of cohort entry and thus would not reflect differences in costs for the index prescription.

### Primary Outcomes

The crude incidence of first moderate or severe COPD exacerbations was 584.4 per 1000 person-years among those receiving fluticasone furoate–vilanterol or budesonide-formoterol, 596.4 per 1000 person-years among those receiving fluticasone furoate–vilanterol or fluticasone propionate–salmeterol, and 589.4 per 1000 person-years among those receiving fluticasone propionate–salmeterol or budesonide-formoterol. Patients receiving fluticasone furoate–vilanterol had a 9% lower risk of experiencing a first moderate or severe COPD exacerbation compared with patients receiving budesonide-formoterol (HR, 0.91 [95% CI, 0.88-0.94]; NNT = 40) and a 6% lower risk of experiencing a first moderate or severe COPD exacerbation compared with patients receiving fluticasone propionate–salmeterol (HR, 0.94 [95% CI, 0.89-0.98]; NNT = 40) (Table 2). eTable 3 in Supplement 1 provides results for the unmatched cohort. The risks of first moderate or severe COPD exacerbation were similar between patients receiving fluticasone propionate–salmeterol and budesonide-formoterol (HR, 0.98 [95% CI, 0.95-1.01]).

**Table 2.** COPD Exacerbations, Pneumonia Hospitalizations, and All-Cause Mortality in Patients Receiving Single-Inhaler Dual Therapy in the Matched Cohort^a^

Outcome	No. of referent events	No. of exposure events	No. of referent events per 1000 person-years	No. of exposure events per 1000 person-years	HR (95% CI)	Risk difference at 365 d (95% CI)	NNT^b^
FF-V (exposure) vs B-F (referent) (n = 38 070 matched pairs)							
Moderate or severe COPD exacerbation	7286	7220	618.7	553.4	0.91 (0.88-0.94)	−2.5 (−3.7 to −1.3)	40
Moderate COPD exacerbation	6595	6515	555.0	494.8	0.91 (0.88-0.94)	−2.2 (−3.6 to −0.8)	45
Severe COPD exacerbation	900	849	66.8	57.1	0.88 (0.80-0.96)	−1.2 (−1.8 to −0.5)	86
Pneumonia hospitalization	1493	1655	112.1	112.8	1.03 (0.96-1.11)	0 (−1.4 to 1.4)	NA
All-cause mortality	1273	1315	93.5	87.5	0.95 (0.88-1.03)	−0.8 (−1.6 to 0.0)	NA
FF-V (exposure) vs FP-S (referent) (n = 20 471 matched pairs)							
Moderate or severe COPD exacerbation	3654	3929	628.9	569.1	0.94 (0.89-0.98)	−2.5 (−4.7 to −0.3)	40
Moderate COPD exacerbation	3202	3490	545.2	500.3	0.95 (0.90-0.99)	−1.7 (−3.9 to 0.5)	NA
Severe COPD exacerbation	557	531	85.2	67.2	0.83 (0.74-0.94)	−1.7 (−2.8 to −0.6)	59
Pneumonia hospitalization	825	879	127.6	112.8	0.93 (0.85-1.03)	−1.4 (−2.6 to −0.1)	73
All-cause mortality	587	624	88.7	78.1	0.90 (0.80-1.01)	−0.4 (−1.5 to 0.7)	NA
FP-S (exposure) vs B-F (referent) (n = 55 627 matched pairs)							
Moderate or severe COPD exacerbation	9872	9390	593.8	584.8	0.98 (0.95-1.01)	−1.5 (−2.8 to −0.1)	69
Moderate COPD exacerbation	8561	8130	508.3	500.4	0.98 (0.95-1.01)	−1.0 (−2.3 to 0.4)	NA
Severe COPD exacerbation	1643	1583	87.7	88.0	0.99 (0.93-1.07)	−0.5 (−1.3 to 0.2)	NA
Pneumonia hospitalization	2179	2184	117.2	122.8	1.04 (0.98-1.10)	0.2 (−0.6 to 1.0)	NA
All-cause mortality	1605	1560	84.4	85.6	1.01 (0.94-1.08)	−0.2 (−0.9 to 0.5)	NA

Abbreviations B-F, budesonide-formoterol; COPD, chronic obstructive pulmonary disease; FF-V, fluticasone furoate–vilanterol; FP-S, fluticasone propionate–salmeterol; HR, hazard ratio; NA, not applicable; NNT, number needed to treat.

^a^ eTable 3 in Supplement 1 reports outcomes in the unmatched cohort.

^b^ Calculated as 1/risk difference at 365 days of follow-up. NNT was calculated only when the 95% CIs for the risk difference excluded the null.

The crude incidence of first pneumonia hospitalization in the matched cohorts was 112.5 per 1000 person-years among those receiving fluticasone furoate–vilanterol or budesonide-formoterol, 119.5 per 1000 person-years among those receiving fluticasone furoate–vilanterol or fluticasone propionate–salmeterol, and 119.9 per 1000 person-years among those receiving fluticasone propionate–salmeterol or budesonide-formoterol. After propensity score matching, a similar risk of pneumonia hospitalization was observed in pairwise comparisons across the 3 cohorts: fluticasone furoate–vilanterol vs budesonide-formoterol (HR, 1.03 [95% CI, 0.96-1.11]), fluticasone furoate–vilanterol vs fluticasone propionate–salmeterol (HR, 0.93 [95% CI, 0.85-1.03]), and fluticasone propionate–salmeterol vs budesonide-formoterol (HR, 1.04 [95% CI, 0.98-1.10]).

**Figure 1.**
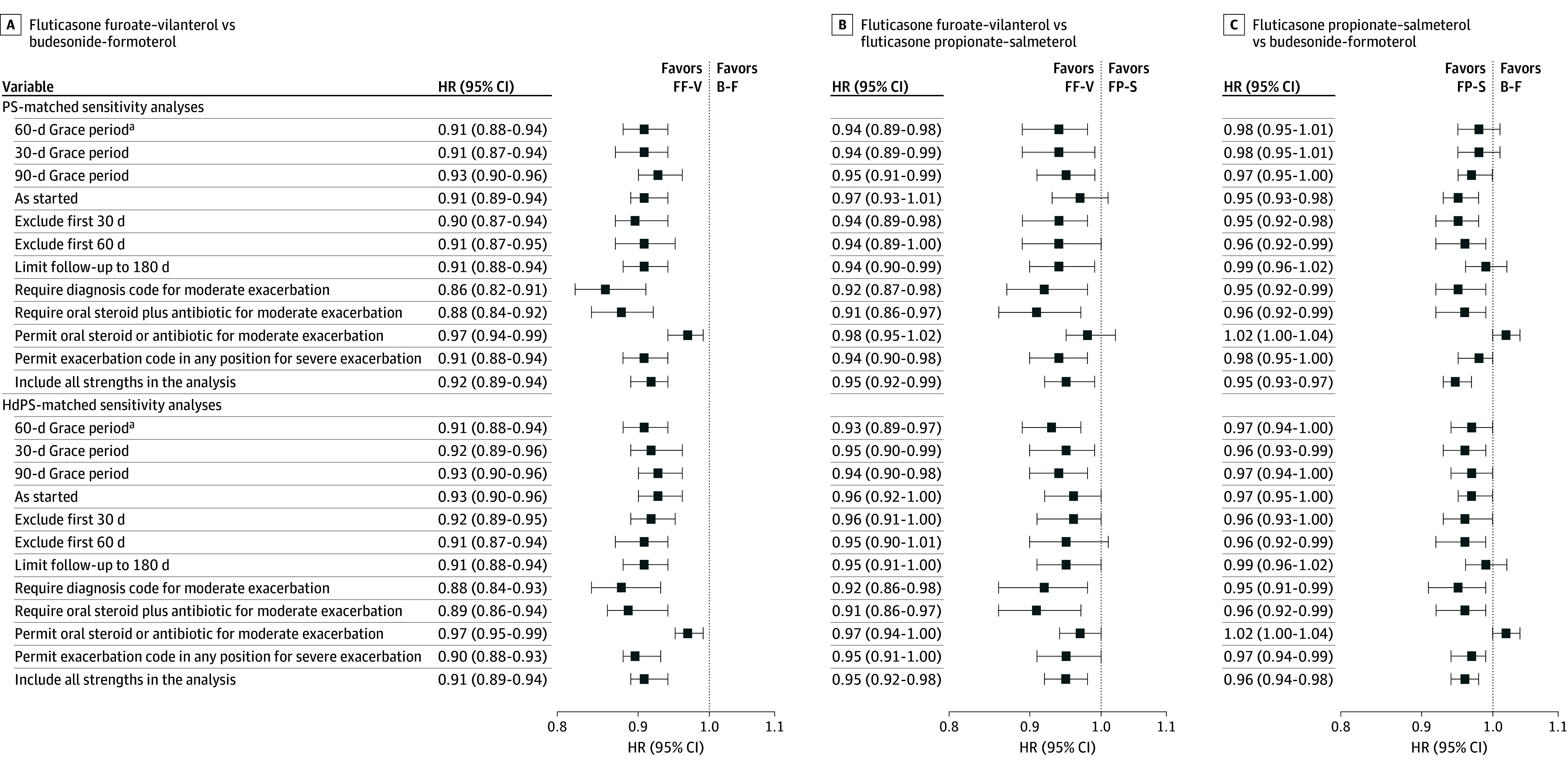
Forest Plot of Sensitivity Analyses for First Moderate or Severe Chronic Obstructive Pulmonary Disease (COPD) Exacerbation Hazard ratios (HRs) and 95% CIs for first moderate or severe COPD exacerbation in new users of the following combination inhaled corticosteroid–long-acting β-agonists under investigation across a range of prespecified sensitivity analyses: fluticasone furoate–vilanterol (FF-V) vs budesonide-formoterol (B-F) (A), FF-V vs fluticasone propionate–salmeterol (FP-S) (B), and FP-S vs B-F (C). ^a^Primary analysis. HdPS indicates high-dimensional propensity score; PS, propensity score.

Reasons for censoring and time to follow-up are given in eTable 4 in Supplement 1 for COPD exacerbations and eTable 5 in Supplement 1 for pneumonia hospitalizations. Kaplan-Meier curves showed constant effects over time for both COPD exacerbations (eFigure 4 in Supplement 1) and pneumonia hospitalizations (eFigure 5 in Supplement 1). Sensitivity analyses of first moderate or severe COPD exacerbation (Figure 1) and first pneumonia hospitalization (eFigure 6 in Supplement 1) yielded findings similar to the primary analyses.

### Moderate vs Severe COPD Exacerbations and All-Cause Mortality

When moderate COPD exacerbations (eFigure 7 in Supplement 1) and severe COPD exacerbations (eFigure 8 in Supplement 1) were analyzed separately, fluticasone furoate–vilanterol was associated with improved outcomes compared with budesonide-formoterol and fluticasone propionate–salmeterol (with the latter 2 associated with similar clinical outcomes to one other). However, the magnitude of observed differences was higher when examining severe COPD exacerbations. Fluticasone furoate–vilanterol was associated with a 12% reduction in the risk of severe COPD exacerbations compared with budesonide-formoterol (HR, 0.88 [95% CI, 0.80-0.96]; NNT = 86) and a 17% reduction compared with fluticasone propionate–salmeterol (HR, 0.83 [95% CI, 0.74-0.94]; NNT = 59). No differences were observed in the incidence of severe COPD exacerbations among patients receiving fluticasone propionate–salmeterol compared with budesonide-formoterol (HR, 0.99 [95% CI, 0.93-1.07]). Rates of all-cause mortality were similar in all 3 pairwise comparisons. Sensitivity analyses for these secondary end points are given in eFigure 7 (moderate COPD exacerbation), eFigure 8 (severe COPD exacerbation), and eFigure 9 (all-cause mortality) in Supplement 1.

### Annual Rates of Events

After 1 year of follow-up, when assessing the overall rate of events (rather than the time to first event), patients receiving fluticasone furoate–vilanterol had 12% fewer moderate or severe COPD exacerbations (incidence rate ratio [IRR], 0.88 [95% CI, 0.85-0.91]) than those receiving budesonide-formoterol and 5% fewer moderate or severe COPD exacerbations (IRR, 0.95 [95% CI, 0.91-0.99]) than those receiving fluticasone propionate–salmeterol. Patients receiving fluticasone propionate–salmeterol had 4% fewer moderate or severe COPD exacerbations compared with those receiving budesonide-formoterol (IRR, 0.96 [95% CI, 0.93-0.99]).

No differences were observed in the rates of pneumonia hospitalization across the 3 cohorts: fluticasone furoate–vilanterol vs budesonide-formoterol (IRR, 1.01 [95% CI, 0.94-1.10]), fluticasone furoate–vilanterol vs fluticasone propionate–salmeterol (IRR, 0.96 [95% CI, 0.86-1.07]), or fluticasone propionate–salmeterol vs budesonide-formoterol (IRR, 1.05 [95% CI, 0.99-1.13]).

### Subgroup Analysis

**Figure 2.**
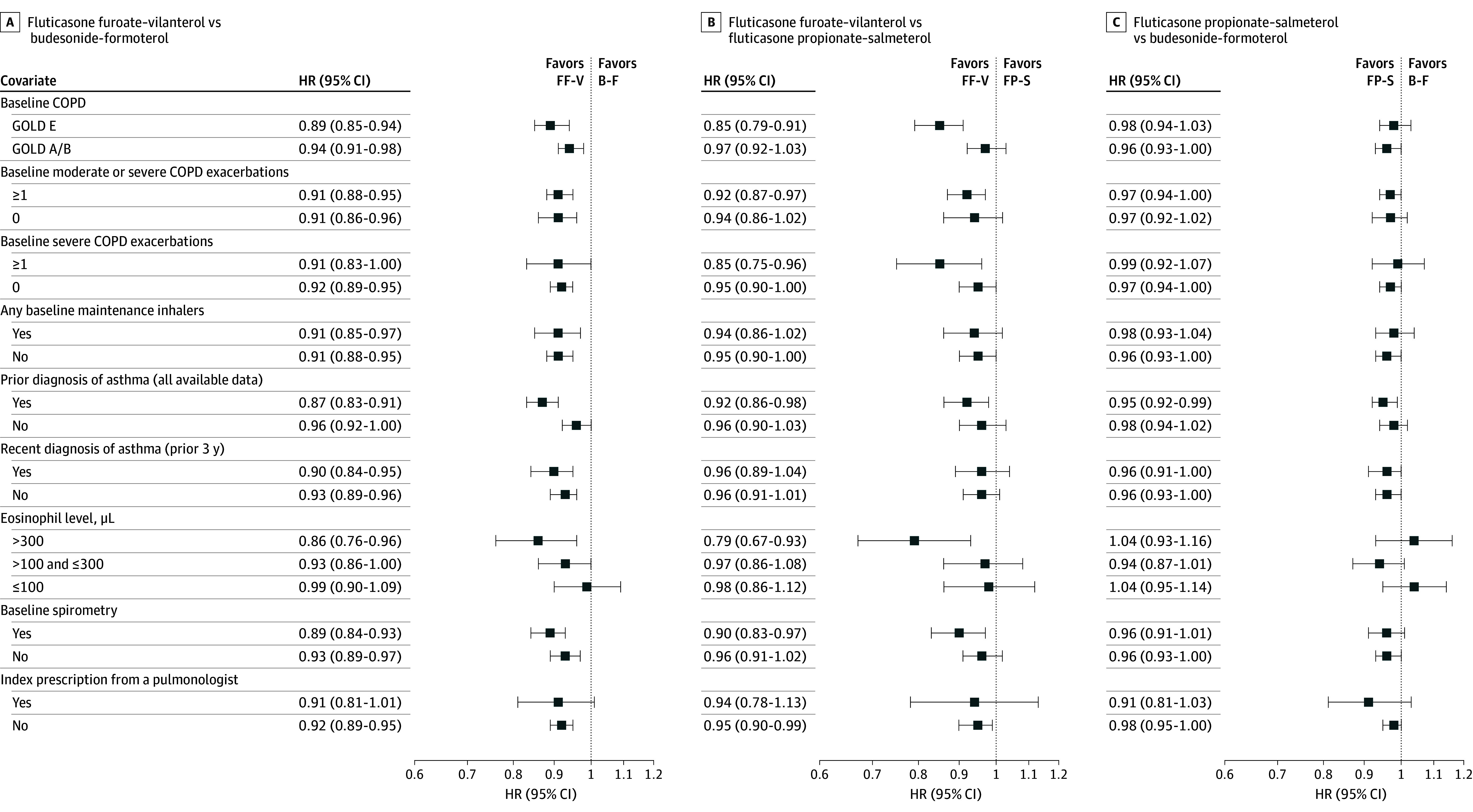
Forest Plot of Subgroup Analyses for First Moderate or Severe Chronic Obstructive Pulmonary Disease (COPD) Exacerbation Hazard ratios (HRs) and 95% CIs for first moderate or severe COPD exacerbation in new users of the following combination inhaled corticosteroid–long-acting β-agonists under investigation across a range of prespecified subgroup analyses: fluticasone furoate–vilanterol (FF-V) vs budesonide-formoterol (B-F) (A), FF-V vs FP-S (B), and FP-S vs B-F (C). GOLD indicates Global Obstructive Lung Disease.

Fluticasone furoate–vilanterol was associated with a lower risk of first moderate or severe COPD exacerbation compared with budesonide-formoterol and fluticasone propionate–salmeterol across most subgroups under investigation, although the 95% CIs were wide in some cases given the small sample sizes (Figure 2). Improved outcomes for fluticasone furoate–vilanterol in both cohorts were observed in those with more severe baseline COPD, including patients with GOLD Group E disease, at least 1 prior moderate or severe COPD exacerbation, high eosinophil counts, and receipt of spirometry during the baseline period. The risks of moderate or severe COPD exacerbations among patients receiving budesonide-formoterol vs fluticasone propionate–salmeterol were similar across subgroups. Similar hazards of first pneumonia hospitalization were observed across subgroups in the 3 cohorts (eFigure 10 in Supplement 1).

## Discussion

In this study, the once-daily dry powder inhaler fluticasone furoate–vilanterol was associated with a similar or slightly reduced risk of moderate or severe COPD exacerbations and no increased risk of pneumonia hospitalizations compared with the twice-daily metered-dose inhaler budesonide-formoterol and the twice-daily dry powder inhaler fluticasone propionate–salmeterol. The magnitude of observed benefit for fluticasone furoate–vilanterol was more pronounced for the outcome of severe COPD exacerbations, with 12% and 17% reductions compared with budesonide-formoterol and fluticasone propionate–salmeterol, respectively. Clinical outcomes for patients receiving fluticasone propionate–salmeterol were similar for patients receiving budesonide-formoterol, although the former was associated with a slightly lower annual rate of moderate or severe COPD exacerbations. The results of prespecified sensitivity, secondary, and subgroup analyses were generally consistent with the findings in our primary analysis across the 3 cohorts.

This research contributes to a growing body of literature on potential intraclass differences among inhalers in the treatment of COPD. One reason why fluticasone furoate–vilanterol could have been associated with small improvements in outcomes compared with twice-daily maintenance inhalers is the convenience of once-daily dosing. Although the analysis used an on-treatment design, censoring patients who discontinued (based on the dispensation date plus days’ supply and a grace period), those using twice-daily inhalers may have been less adherent to the dosing regimen, leading to reduced effectiveness. Another potential explanation concerns the delivery device itself; a prior study found that patients using Ellipta inhalers (in which fluticasone furoate–vilanterol is sold) made fewer errors than patients using metered-dose inhalers (in which budesonide-formoterol is sold) or the dry powder Diskus inhaler (in which fluticasone propionate–salmeterol is sold).^39^ Both hypotheses are consistent with a 2024 study of triple inhaler therapy finding that once-daily fluticasone furoate–umeclidinium–vilanterol dry powder Ellipta inhalers were associated with slightly reduced risks of moderate or severe COPD exacerbations and similar risks of pneumonia hospitalization compared with twice-daily budesonide–glycopyrrolate–formoterol metered-dose inhalers.^19^ Our findings add further support to the possibility that dosing frequency and/or device type could contribute to small intraclass differences among ICS-containing maintenance inhalers in the treatment of COPD.

Another potential explanation for the small observed benefit of fluticasone furoate–vilanterol concerns the active moieties. Fluticasone furoate and fluticasone propionate may have subtly different effects on the airways compared with each other and with budesonide; all 3 inhalers under investigation also employed different LABAs (vilanterol, formoterol, and salmeterol), which could have been associated with varying degrees of effectiveness and safety. Further research is needed to explore potential intraclass differences among active pharmaceutical compounds in the treatment of COPD.

The present study also contributes to growing research showing that dry powder maintenance inhalers, which have environmental advantages over metered-dose formulations, appear to be no worse for patients than higher-emission products in the same therapeutic class.^3,19,40^ Indeed, the once-daily dry powder ICS-LABA in our study was associated with improved clinical outcomes, including reduced COPD hospitalizations, which are themselves emissions generating. Our findings differ from a recent study of patients in the Veterans Affairs (VA) health system, which found that switching from budesonide-formoterol to fluticasone-salmeterol was associated with slightly worse clinical ouctomes.^41^ However, the superiority of budesonide-formoterol in that study could be attributed to challenges with switching rather than the products themselves. Our study of new users, by contrast, excluded patients who received ICS-LABAs in the 365 days before cohort entry to avoid concerns related to switching. Further research is needed to investigate patients who switch from metered-dose to dry powder inhalers in cohorts outside the VA health system.

### Limitations

This study has some limitations. First, although we controlled for many covariates, the possibility of residual confounding cannot be excluded. Given the limitations of claims-based analyses, we could not adjust for several factors of importance, such as forced expiratory volume in 1 second and insurance plan type. Observational studies of metered-dose vs dry powder inhalers raise particular questions about bias from selective prescribing, since metered-dose inhalers are considered unsuitable by some clinicians for patients who have poor inspiratory force. However, formularies often cover only 1 or 2 inhalers in a given class, reducing concerns about this type of bias. Additionally, sensitivity analyses using high-dimensional propensity score matching, which balance for hundreds of covariates that can serve as proxies for unmeasured confounders,^32,33,42^ yielded similar findings to the primary analysis. Nonetheless, the possibility of residual confounding remains as an explanation for our findings. Second, despite using a validated algorithm to identify patients with COPD, some patients may have been misclassified, which could have biased findings toward the null hypothesis. Third, many patients discontinued treatment; thus, follow-up was short. However, the as-started sensitivity analyses, which followed patients even after product discontinuation or switching, generated findings that were similar to the primary analysis. Fourth, although the exposure and referent groups within each cohort were well balanced after matching, there were subtle differences in the features of participants across cohorts, which may limit generalizability. Finally, in the most recent GOLD guidelines, ICS-LAMA-LABAs are now favored over ICS-LABAs for patients who require an inhaled corticosteroid.^5^ However, use of ICS-LABAs remains widespread, and understanding potential intraclass differences among these products therefore remains important.

## Conclusions

In this cohort study of ICS-LABA therapy in patients with COPD, fluticasone furoate–vilanterol was associated with similar or slightly improved clinical outcomes compared with budesonide-formoterol and fluticasone propionate–salmeterol. Further studies are needed to explore potential intraclass differences among inhalers in the treatment of COPD. These findings can help inform physician prescribing choices, clinical guidelines, insurance formulary design, reimbursement decisions, and health care sustainability initiatives.

## Notes

Supplement 1.
eMethods
**eTable 1.** Inclusion and Exclusion Criteria **eTable 2.** Baseline Characteristics of Patients in the Unmatched Cohorts Initiating ICS-LABA Therapy **eTable 3.** COPD Exacerbations, Pneumonia Hospitalizations, and All-Cause Mortality in Patients Receiving Single-Inhaler Dual Therapy in the Unmatched Cohort **eTable 4.** Reasons for Censoring in the Analysis of First Moderate or Severe COPD Exacerbation **eTable 5.** Reasons for Censoring in the Analysis of First Pneumonia Hospitalization **eFigure 1.** Study Design Comparing New Users of ICS-LABAs Included in the Analysis **eFigure 2.** Cohort Composition **eFigure 3.** Propensity Score Distributions Before and After Matching **eFigure 4.** Kaplan-Meier Plots for First Moderate or Severe COPD Exacerbation **eFigure 5.** Kaplan-Meier Plots for First Pneumonia Hospitalization **eFigure 6.** Sensitivity Analysis for First Pneumonia Hospitalization **eFigure 7.** Sensitivity Analysis for First Moderate COPD Exacerbation **eFigure 8.** Sensitivity Analysis for First Severe COPD Exacerbation **eFigure 9.** Sensitivity Analysis for All-Cause Mortality **eFigure 10.** Subgroup Analysis for First Pneumonia Hospitalization
eReferences

Supplement 2.
Data Sharing Statement
