# Supplementary material for: Fluticasone- vs Budesonide-Based Dual Therapy for COPD
Source: JAMA Netw Open. 2026 Mar 9;9(3):e260959. doi: 10.1001/jamanetworkopen.2026.0959 (PMC12973110; doi:10.1001/jamanetworkopen.2026.0959)
Supplement: Supplement 1. — eMethods eTable 1. Inclusion and Exclusion Criteria eTable 2. Baseline Characteristics of Patients in the Unmatched Cohorts Initiating ICS-LABA Therapy eTable 3. COPD Exacerbations, Pneumonia Hospitalizations, and All-Cause Mortality in Patients Receiving Single-Inhaler Dual Therapy in the Unmatched Cohort eTable 4. Reasons for Censoring in the Analysis of First Moderate or Severe COPD Exacerbation eTable 5. Reasons for Censoring in the Analysis of First Pneumonia Hospitalization eFigure 1. Study Design Comparing New Users of ICS-LABAs Included in the Analysis eFigure 2. Cohort Composition eFigure 3. Propensity Score Distributions Before and After Matching eFigure 4. Kaplan-Meier Plots for First Moderate or Severe COPD Exacerbation eFigure 5. Kaplan-Meier Plots for First Pneumonia Hospitalization eFigure 6. Sensitivity Analysis for First Pneumonia Hospitalization eFigure 7. Sensitivity Analysis for First Moderate COPD Exacerbation eFigure 8. Sensitivity Analysis for First Severe COPD Exacerbation eFigure 9. Sensitivity Analysis for All-Cause Mortality eFigure 10. Subgroup Analysis for First Pneumonia Hospitalization eReferences [file jamanetwopen-e260959-s001.pdf]

Supplemental Online Content

Feldman WB, Ambati VL, Suissa S, et al. Fluticasone- vs budesonide-based dual therapy for COPD. *JAMA Netw Open*. 2026;9(3):e260959. doi:10.1001/jamanetworkopen.2026.0959

eMethods

eTable 1. Inclusion and Exclusion Criteria

eTable 2. Baseline Characteristics of Patients in the Unmatched Cohorts Initiating ICS-LABA Therapy

eTable 3. COPD Exacerbations, Pneumonia Hospitalizations, and All-Cause Mortality in Patients Receiving Single-Inhaler Dual Therapy in the Unmatched Cohort

eTable 4. Reasons for Censoring in the Analysis of First Moderate or Severe COPD Exacerbation

eTable 5. Reasons for Censoring in the Analysis of First Pneumonia Hospitalization

eFigure 1. Study Design Comparing New Users of ICS-LABAs Included in the Analysis

eFigure 2. Cohort Composition

eFigure 3. Propensity Score Distributions Before and After Matching

eFigure 4. Kaplan-Meier Plots for First Moderate or Severe COPD Exacerbation

eFigure 5. Kaplan-Meier Plots for First Pneumonia Hospitalization

eFigure 6. Sensitivity Analysis for First Pneumonia Hospitalization

eFigure 7. Sensitivity Analysis for First Moderate COPD Exacerbation

eFigure 8. Sensitivity Analysis for First Severe COPD Exacerbation

eFigure 9. Sensitivity Analysis for All-Cause Mortality

eFigure 10. Subgroup Analysis for First Pneumonia Hospitalization

eReferences

This supplemental material has been provided by the authors to give readers additional information about their work.

© 2026 Feldman WB et al. *JAMA Network Open*.

eMethods

<u>Building study cohorts</u>

Use of a given inhaler was identified according to the brand-name listed on the associated insurance claim. Two inhalers have three approved strengths (fluticasone furoate-vilanterol and fluticasone propionate-salmeterol), while one inhaler has two approved strengths (budesonide-formoterol). Strengths were determined from the insurance claims. For each pairwise comparison, we set the start of each study period to align with the first full year that both products under investigation were on the US market. Patients with co-occurring asthma diagnoses were included to enhance generalizability, but separate subgroup analyses were performed among patients with chronic obstructive pulmonary disease (COPD) alone. Patients entered a given cohort on the first treatment episode that met all eligibility criteria; patients could only enter each of the three cohorts once (in either the exposure or referent group). However, they could enter more than one cohort; for example, a new user of fluticasone furoate-vilanterol could have been included in cohort #1 (comparing new users of fluticasone furoate-vilanterol vs. budesonide-formoterol) and in cohort #2 (comparing new users of fluticasone furoate-vilanterol vs. fluticasone propionate-salmeterol).

<u>Covariates included in the propensity score model</u>

We included all of the following covariates, which were measured in the 365 days before cohort entry, in our propensity score model: (1) measures of baseline lung disease: moderate COPD exacerbations, severe COPD exacerbations, Global Initiative for Chronic Obstructive Lung Disease (GOLD) Group E severity,[1] pneumonia hospitalizations, fills of maintenance and rescue inhalers, respiratory antibiotics, chronic azithromycin, roflumilast, chronic prednisone use, claims for home oxygen or oxygen equipment, use of continuous or bilevel positive airway

© 2026 Feldman WB et al. *JAMA Network Open*.

pressure machines, smoking, and pulmonary rehabilitation; (2) other co-morbidities: combined comorbidity score,[2] frailty score,[3] obstructive sleep apnea, hypertension, diabetes, obesity, coronary artery disease, peripheral vascular disease, venous thromboembolic disease, congestive heart failure, gastroesophageal reflux disease, renal failure, osteoporosis, dementia or other neurologic disease, malignancy, anxiety disorder, and depression; (3) healthcare utilization: emergency department visits, hospitalizations, 90-day readmissions, office visits, pulmonology visits, total number of prescription drug claims, basic or comprehensive metabolic panels, electrocardiograms, echocardiograms, CT scans, bronchoscopies, colonoscopies, mammography, bone mineral density scans, and influenza vaccinations; and (4) use of other medications: statins, beta-blockers, angiotensin-converting enzyme inhibitors, angiotensin-receptor blockers, calcium channel blockers, thiazide diuretics, loop diuretics, proton-pump inhibitors, H2-receptor blockers, metformin, sulfonylureas, sodium-glucose cotransporter-2 inhibitors, dipeptidyl-peptidase IV inhibitors, glucagon-like peptide-1 inhibitors, benzodiazepines, and selective serotonin reuptake or serotonin-norepinephrine reuptake inhibitors.

Defining outcomes

Moderate exacerbations were defined by prescription fills of oral glucocorticoids, and severe exacerbations were defined by hospitalizations for COPD. In some cases, patients may first receive oral steroids before being admitted to the hospital; in other cases, a patient may be discharged after a hospitalization with a script for oral steroids. When either of these scenarios occurred, we considered the exacerbation to be severe and to have begun on the first day when criteria for the exacerbation (moderate or severe) were first met.

© 2026 Feldman WB et al. *JAMA Network Open*.

Subgroup analyses

We analyzed patients in several subgroups of interest, with separate analyses performed for those meeting subgroup criteria and those who did not meet such criteria: (1) baseline GOLD Group E disease, defined as having at least one severe or two moderate exacerbations in the year before cohort entry; (2) at least one moderate or severe COPD exacerbation during the baseline assessment period; (3) at least one severe COPD exacerbation during the baseline assessment period; (4) use of any maintenance inhaler during the baseline assessment period; (5) prior diagnosis code for asthma (using all available data); (6) active diagnosis of asthma (based on at least 3 outpatient claims or inpatient claim for asthma in the 3 years before cohort entry); (7) eosinophil levels > 300/microliter; (8) spirometry during the baseline assessment period; and (9) receipt of their index prescription from a pulmonologist.

© 2026 Feldman WB et al. *JAMA Network Open*.

eTable 1. Inclusion and Exclusion Criteria

**Inclusion criteria**
Initiation of exposure or referent[a] product during the study period[b]
Enrollment in the dataset for at least 365 days
A diagnosis of COPD, defined as at least 3 outpatient or inpatient diagnosis codes in the 3 years before cohort entry (ICD-9-CM codes incldue 491.xx, 492.xx, or 496, and ICD-10-CM codes include J41.x, J42.x, J43.x, J44.x) (PPV 0.82)[4]
Age ≥ 40 to increase the specificity of the COPD diagnosis
**Exclusion criteria**
Receipt of any ICS-LABA (or separate ICS + LABA within 30 days of each other) during the 365 days before cohort entry (until the day before cohort entry)
Receipt of any ICS-LABA besides the exposure or reference product on the cohort entry date (patients who received both the exposure and reference product were excluded)
Receipt of any other maintenance inhaler on the cohort entry date

COPD: Chronic obstructive pulmonary disease; ICD-9-CM: International Classification of Diseases, Ninth Revision, Clinical Modification; ICD-10-CM: International Classification of Diseases, Tenth Revision, Clinical Modification; PPV: positive predictive value; ICS: inhaled corticosteroid; LABA: long-acting beta-agonist

a. The exposures and referent drugs differed across the 3 studies: (1) Study #1: fluticasone furoate-vilanterol (exposure) versus fluticasone propionate-salmeterol (referent); (2) Study #2: fluticasone furoate-vilanterol (exposure) versus budesonide-formoterol (referent); (3) Study #3: fluticasone propionate-salmeterol (referent) vs. budesonide-formoterol (exposure).

b. The study periods were selected to ensure that both products under investigation were available on the US market throughout: (1) Study #1: January 1, 2014 to February 29, 2024; (2) Study #2: January 1, 2014 to February 29, 2024; and (3) Study #3: January 1, 2007 to February 29, 2024.

© 2026 Feldman WB et al. *JAMA Network Open*.

eTable 2. Baseline Characteristics of Patients in the Unmatched Cohorts Initiating ICS-LABA Therapy

	**Cohort 1**			**Cohort 2**			**Cohort 3**		
	**Fluticasone furoate-vilanterol vs. budesonide-formoterol (2014-2024)**			**Fluticasone furoate-vilanterol vs. fluticasone propionate-salmeterol (2014-2024)**			**Fluticasone propionate-salmeterol vs. budesonide-formoterol (2007-2024)**		
	Budesonide-formoterol (n= 90,883)	Fluticasone furoate-vilanterol (n= 38,164)	Abs. St. Diff.	Fluticasone propionate-salmeterol (n= 39,152)	Fluticasone furoate-vilanterol (n= 38,277)	Abs. St. Diff.	Budesonide-formoterol (n= 110,396)	Fluticasone propionate-salmeterol (n= 88,958)	Abs. St. Diff.
Age, mean (SD)	69.85 (9.18)	71.03 (9.16)	0.128	69.55 (9.10)	71.03 (9.16)	0.162	69.05 (9.18)	67.90 (8.72)	0.128
Gender, n (%)									
Male	39,183 (43.1%)	15,781 (41.4%)	0.036	16,079 (41.1%)	15,833 (41.4%)	0.006	48,402 (43.8%)	38,410 (43.2%)	0.013
Female	51,684 (56.9%)	22,381 (58.6%)	0.036	23,065 (58.9%)	22,442 (58.6%)	0.006	61,975 (56.1%)	50,524 (56.8%)	0.013
Missing	16 (0.0%)	2 (0.0%)	0.012	8 (0.0%)	2 (0.0%)	0.013	19 (0.0%)	24 (0.0%)	0.007
Region, n (%)									
Northeast	9,008 (9.9%)	4,628 (12.1%)	0.071	4,439 (11.3%)	4,647 (12.1%)	0.025	10,714 (9.7%)	8,656 (9.7%)	0.001
Midwest	18,063 (19.9%)	7,613 (19.9%)	0.002	7,191 (18.4%)	7,648 (20.0%)	0.041	22,015 (19.9%)	16,332 (18.4%)	0.040
South	47,043 (51.8%)	19,253 (50.4%)	0.026	16,576 (42.3%)	19,338 (50.5%)	0.165	56,600 (51.3%)	38,003 (42.7%)	0.172
West	16,595 (18.3%)	6,595 (17.3%)	0.026	10,842 (27.7%)	6,571 (17.2%)	0.254	20,845 (18.9%)	25,719 (28.9%)	0.237
Missing	174 (0.2%)	75 (0.2%)	0.001	104 (0.3%)	73 (0.2%)	0.016	222 (0.2%)	248 (0.3%)	0.016
Season of Cohort Entry									
Winter	24,697 (27.2%)	9,904 (26.0%)	0.028	11,081 (28.3%)	9,948 (26.0%)	0.052	29,587 (26.8%)	24,324 (27.3%)	0.012
Spring	24,503 (27.0%)	10,241 (26.8%)	0.003	10,691 (27.3%)	10,271 (26.8%)	0.011	29,222 (26.5%)	23,885 (26.8%)	0.009
Summer	21,156 (23.3%)	9,118 (23.9%)	0.014	8,809 (22.5%)	9,154 (23.9%)	0.034	25,806 (23.4%)	20,380 (22.9%)	0.011
Fall	20,527 (22.6%)	8,901 (23.3%)	0.018	8,571 (21.9%)	8,904 (23.3%)	0.033	25,781 (23.4%)	20,369 (22.9%)	0.011
Year of Cohort Entry									
2007	-	-	-	-	-	-	309 (0.3%)	6,331 (7.1%)	0.368
2008	-	-	-	-	-	-	1,245 (1.1%)	6,461 (7.3%)	0.310
2009	-	-	-	-	-	-	3,019 (2.7%)	7,795 (8.8%)	0.261
2010	-	-	-	-	-	-	4,099 (3.7%)	8,307 (9.3%)	0.229
2011	-	-	-	-	-	-	4,168 (3.8%)	7,851 (8.8%)	0.209
2012	-	-	-	-	-	-	4,905 (4.4%)	8,016 (9.0%)	0.183
2013	-	-	-	-	-	-	5,800 (5.3%)	8,597 (9.7%)	0.168
2014	6,579 (7.2%)	1,279 (3.4%)	0.174	8,062 (20.6%)	1,279 (3.3%)	0.551	5,768 (5.2%)	6,844 (7.7%)	0.101
2015	6,129 (6.7%)	2,376 (6.2%)	0.021	7,179 (18.3%)	2,372 (6.2%)	0.377	5,470 (5.0%)	6,216 (7.0%)	0.086
2016	8,549 (9.4%)	3,363 (8.8%)	0.021	6,346 (16.2%)	3,346 (8.7%)	0.227	7,804 (7.1%)	5,751 (6.5%)	0.024
2017	9,669 (10.6%)	4,862 (12.7%)	0.065	6,647 (17.0%)	4,841 (12.6%)	0.122	9,124 (8.3%)	6,260 (7.0%)	0.046
2018	10,193 (11.2%)	5,255 (13.8%)	0.077	5,739 (14.7%)	5,231 (13.7%)	0.028	9,740 (8.8%)	5,497 (6.2%)	0.100
2019	11,449 (12.6%)	5,032 (13.2%)	0.018	1,786 (4.6%)	5,022 (13.1%)	0.305	11,068 (10.0%)	1,718 (1.9%)	0.346
2020	10,583 (11.6%)	4,171 (10.9%)	0.023	818 (2.1%)	4,186 (10.9%)	0.364	10,347 (9.4%)	792 (0.9%)	0.392
2021	8,952 (9.9%)	4,352 (11.4%)	0.050	858 (2.2%)	4,409 (11.5%)	0.376	8,824 (8.0%)	847 (1.0%)	0.346
2022	8,528 (9.4%)	3,686 (9.7%)	0.009	853 (2.2%)	3,741 (9.8%)	0.325	8,474 (7.7%)	824 (0.9%)	0.337
2023	8,815 (9.7%)	3,237 (8.5%)	0.042	819 (2.1%)	3,290 (8.6%)	0.292	8,795 (8.0%)	807 (0.9%)	0.348
2024	1,437 (1.6%)	551 (1.4%)	0.011	45 (0.1%)	560 (1.5%)	0.153	1,437 (1.3%)	44 (0.0%)	0.153

© 2026 Feldman WB et al. *JAMA Network Open*.

Baseline lung disease									
Baseline GOLD E, n (%)	21,822 (24.0%)	8,447 (22.1%)	0.045	8,821 (22.5%)	8,454 (22.1%)	0.011	26,407 (23.9%)	19,708 (22.2%)	0.042
Moderate COPD exacerbations, mean (SD)	0.76 (1.13)	0.69 (1.05)	0.060	0.66 (1.02)	0.69 (1.05)	0.037	0.75 (1.12)	0.61 (0.98)	0.133
Severe COPD exacerbations, mean (SD)	0.09 (0.33)	0.08 (0.33)	0.017	0.10 (0.36)	0.08 (0.33)	0.067	0.09 (0.34)	0.11 (0.37)	0.069
SABA fills, mean (SD)	2.18 (3.42)	2.02 (3.25)	0.048	2.03 (3.34)	2.01 (3.25)	0.004	2.22 (3.52)	2.03 (3.49)	0.052
SAMA fills, mean (SD)	0.09 (0.76)	0.08 (0.67)	0.020	0.10 (0.80)	0.07 (0.67)	0.033	0.11 (0.87)	0.19 (1.17)	0.070
SAMA-SABA fills, mean (SD)	0.59 (1.97)	0.54 (1.94)	0.022	0.62 (2.14)	0.54 (1.94)	0.036	0.64 (2.12)	0.74 (2.35)	0.043
Pneumonia hospitalizations, mean (SD)	0.13 (0.45)	0.14 (0.48)	0.027	0.14 (0.47)	0.14 (0.48)	0.012	0.13 (0.45)	0.14 (0.47)	0.029
Respiratory antibiotic fills, mean (SD)	1.78 (2.09)	1.68 (2.00)	0.046	1.69 (1.98)	1.68 (1.99)	0.005	1.84 (2.14)	1.79 (2.04)	0.024
Any prior claim for asthma[a], n (%)	42,103 (46.3%)	16,817 (44.1%)	0.045	19,482 (49.8%)	16,836 (44.0%)	0.116	51,538 (46.7%)	42,434 (47.7%)	0.020
Home oxygen or equipment claim, n (%)	20,987 (23.1%)	8,137 (21.3%)	0.043	8,802 (22.5%)	8,131 (21.2%)	0.030	25,742 (23.3%)	20,755 (23.3%)	<0.001
CPAP or BiPAP, n (%)	9,300 (10.2%)	4,008 (10.5%)	0.009	3,112 (7.9%)	4,031 (10.5%)	0.089	10,955 (9.9%)	6,369 (7.2%)	0.099
Spirometry, n (%)	26,905 (29.6%)	14,338 (37.6%)	0.169	10,064 (25.7%)	14,385 (37.6%)	0.257	36,016 (32.6%)	27,484 (30.9%)	0.037
Index prescription by pulmonologist, n (%)	5,319 (5.9%)	3,551 (9.3%)	0.131	1,557 (4.0%)	3,567 (9.3%)	0.216	6,898 (6.2%)	4,286 (4.8%)	0.063
Smoking, n (%)	52,644 (57.9%)	22,407 (58.7%)	0.016	19,886 (50.8%)	22,486 (58.7%)	0.160	59,491 (53.9%)	37,863 (42.6%)	0.228
Pulmonary rehabilitation, n (%)	629 (0.7%)	264 (0.7%)	<0.001	169 (0.4%)	268 (0.7%)	0.036	805 (0.7%)	495 (0.6%)	0.022
LAMA, n (%)	13,088 (14.4%)	5,558 (14.6%)	0.005	6,384 (16.3%)	5,567 (14.5%)	0.049	18,161 (16.5%)	17,142 (19.3%)	0.074
LABA, n (%)	968 (1.1%)	446 (1.2%)	0.010	441 (1.1%)	448 (1.2%)	0.004	2,025 (1.8%)	1,669 (1.9%)	0.003
ICS, n (%)	6,181 (6.8%)	2,821 (7.4%)	0.023	2,522 (6.4%)	2,825 (7.4%)	0.037	9,258 (8.4%)	7,562 (8.5%)	0.004
LAMA-LABA, n (%)	3,193 (3.5%)	1,870 (4.9%)	0.069	620 (1.6%)	1,880 (4.9%)	0.189	3,101 (2.8%)	590 (0.7%)	0.165
ICS-LAMA-LABA, n (%)	2,840 (3.1%)	1,089 (2.9%)	0.016	187 (0.5%)	1,115 (2.9%)	0.189	2,814 (2.5%)	178 (0.2%)	0.203
Any maintenance inhaler, n (%)	22,793 (25.1%)	10,175 (26.7%)	0.036	8,993 (23.0%)	10,214 (26.7%)	0.086	30,097 (27.3%)	23,684 (26.6%)	0.014
Chronic azithromycin, n (%)	761 (0.8%)	239 (0.6%)	0.025	180 (0.5%)	239 (0.6%)	0.022	852 (0.8%)	352 (0.4%)	0.049
Roflumilast, n (%)	550 (0.6%)	154 (0.4%)	0.028	150 (0.4%)	156 (0.4%)	0.004	587 (0.5%)	204 (0.2%)	0.049
Chronic oral steroids, n (%)	6,616 (7.3%)	2,539 (6.7%)	0.025	2,549 (6.5%)	2,533 (6.6%)	0.004	8,609 (7.8%)	6,865 (7.7%)	0.003
Events within 30 days of cohort entry, n (%)									
Moderate or severe COPD exacerbation	19,194 (21.1%)	6,910 (18.1%)	0.076	8,900 (22.7%)	6,889 (18.0%)	0.118	23,041 (20.9%)	19,892 (22.4%)	0.036
Respiratory antibiotic fill	24,149 (26.6%)	8,739 (22.9%)	0.085	11,030 (28.2%)	8,759 (22.9%)	0.122	29,981 (27.2%)	26,845 (30.2%)	0.067
Baseline eosinophils, n (%)									
CBC with differential performed	55,610 (61.2%)	24,272 (63.6%)	0.050	22,165 (56.6%)	24,351 (63.6%)	0.143	65,440 (59.3%)	46,447 (52.2%)	0.143
Eosinophil categories									
Eosinophils ≤ 100/microliter	7,966 (8.8%)	3,456 (9.1%)	0.010	3,216 (8.2%)	3,469 (9.1%)	0.030	8,885 (8.0%)	5,334 (6.0%)	0.080
Eosinophils > 100 and ≤ 300/microliter	11,167 (12.3%)	4,957 (13.0%)	0.021	4,475 (11.4%)	4,961 (13.0%)	0.047	12,211 (11.1%)	6,863 (7.7%)	0.115
Eosinophils > 300/microliter	4,764 (5.2%)	2,125 (5.6%)	0.014	1,893 (4.8%)	2,133 (5.6%)	0.033	5,248 (4.8%)	2,864 (3.2%)	0.078
Missing	66,986 (73.7%)	27,626 (72.4%)	0.030	29,568 (75.5%)	27,714 (72.4%)	0.071	84,052 (76.1%)	73,897 (83.1%)	0.173
Other co-morbidities									
Combined comorbidity index, mean (SD)	3.95 (3.24)	4.27 (3.41)	0.095	3.80 (3.20)	4.26 (3.41)	0.141	3.70 (3.17)	3.36 (3.01)	0.109
Frailty score, mean (SD)	0.21 (0.07)	0.21 (0.08)	0.042	0.21 (0.08)	0.21 (0.08)	0.034	0.21 (0.07)	0.21 (0.07)	0.001
Obstructive sleep apnea, n (%)	19,144 (21.1%)	8,521 (22.3%)	0.031	6,414 (16.4%)	8,566 (22.4%)	0.152	21,666 (19.6%)	11,456 (12.9%)	0.184
Hypertension, n (%)	74,550 (82.0%)	31,398 (82.3%)	0.006	31,144 (79.5%)	31,518 (82.3%)	0.071	89,025 (80.6%)	68,599 (77.1%)	0.087
Diabetes, n (%)	34,667 (38.1%)	14,020 (36.7%)	0.029	14,333 (36.6%)	14,080 (36.8%)	0.004	40,442 (36.6%)	29,833 (33.5%)	0.065
Obesity, n (%)	24,291 (26.7%)	10,349 (27.1%)	0.009	8,595 (22.0%)	10,390 (27.1%)	0.121	26,357 (23.9%)	14,076 (15.8%)	0.203
Coronary artery disease, n (%)	33,024 (36.3%)	14,560 (38.2%)	0.038	13,281 (33.9%)	14,614 (38.2%)	0.089	39,259 (35.6%)	30,323 (34.1%)	0.031

© 2026 Feldman WB et al. *JAMA Network Open*.

Peripheral vascular disease, n (%)	26,703 (29.4%)	12,005 (31.5%)	0.045	11,085 (28.3%)	12,030 (31.4%)	0.068	30,391 (27.5%)	21,956 (24.7%)	0.065
Venous thromboembolic disease, n (%)	3,904 (4.3%)	1,775 (4.7%)	0.017	1,937 (4.9%)	1,779 (4.6%)	0.014	4,943 (4.5%)	4,898 (5.5%)	0.047
Congestive heart failure, n (%)	25,363 (27.9%)	11,272 (29.5%)	0.036	10,862 (27.7%)	11,288 (29.5%)	0.039	30,024 (27.2%)	25,056 (28.2%)	0.022
Gatroesophageal reflux disease, n (%)	32,296 (35.5%)	13,930 (36.5%)	0.020	12,269 (31.3%)	13,992 (36.6%)	0.110	37,444 (33.9%)	24,550 (27.6%)	0.137
Renal failure, n (%)	19,932 (21.9%)	9,222 (24.2%)	0.053	9,305 (23.8%)	9,216 (24.1%)	0.007	22,364 (20.3%)	17,438 (19.6%)	0.016
Osteoporosis, n (%)	8,528 (9.4%)	4,106 (10.8%)	0.046	3,684 (9.4%)	4,100 (10.7%)	0.043	10,115 (9.2%)	8,181 (9.2%)	0.001
Dementia/other neurologic disease, n (%)	7,753 (8.5%)	4,027 (10.6%)	0.069	3,073 (7.8%)	4,034 (10.5%)	0.093	7,949 (7.2%)	4,598 (5.2%)	0.084
Malignancy, non-metastatic, n (%)	12,273 (13.5%)	5,499 (14.4%)	0.026	5,143 (13.1%)	5,518 (14.4%)	0.037	15,062 (13.6%)	12,495 (14.0%)	0.012
Metastatic solid organ malignancy, n (%)	2,200 (2.4%)	1,010 (2.6%)	0.014	916 (2.3%)	1,017 (2.7%)	0.020	2,635 (2.4%)	2,183 (2.5%)	0.004
Anxiety disorder, n (%)	25,811 (28.4%)	10,750 (28.2%)	0.005	9,502 (24.3%)	10,830 (28.3%)	0.092	28,407 (25.7%)	16,414 (18.5%)	0.176
Depression, n (%)	25,137 (27.7%)	10,691 (28.0%)	0.008	9,909 (25.3%)	10,719 (28.0%)	0.061	27,734 (25.1%)	17,606 (19.8%)	0.128
Healthcare utilization									
Emergency department visits, mean (SD)	2.80 (5.29)	2.71 (4.64)	0.017	2.84 (7.04)	2.70 (4.56)	0.023	2.66 (5.03)	2.55 (5.52)	0.021
Hospitalizations, mean (SD)	0.70 (1.40)	0.79 (1.54)	0.056	0.78 (1.50)	0.78 (1.54)	<0.001	0.69 (1.37)	0.79 (1.45)	0.070
90-day readmissions, mean (SD)	0.17 (0.74)	0.18 (0.75)	0.011	0.18 (0.76)	0.18 (0.74)	0.001	0.17 (0.73)	0.18 (0.75)	0.015
Office visits, mean (SD)	11.91 (9.02)	12.32 (9.07)	0.046	11.18 (8.84)	12.32 (9.09)	0.127	11.89 (8.99)	10.97 (8.69)	0.103
Pulmonology visits, mean (SD)	0.21 (0.85)	0.28 (0.96)	0.072	0.15 (0.74)	0.28 (0.96)	0.144	0.23 (0.91)	0.17 (0.79)	0.070
Prescription drug claims, mean (SD)	52.36 (38.86)	52.36 (41.79)	<0.001	52.48 (41.54)	52.39 (41.86)		52.25 (38.62)	51.85 (40.05)	0.010
Basic or complete metabolic panel, n (%)	78,286 (86.1%)	33,420 (87.6%)	0.042	32,130 (82.1%)	33,530 (87.6%)	0.155	93,000 (84.2%)	67,797 (76.2%)	0.203
Electrocardiogram, n (%)	58,154 (64.0%)	25,352 (66.4%)	0.051	24,854 (63.5%)	25,441 (66.5%)	0.063	70,291 (63.7%)	57,126 (64.2%)	0.011
Echocardiogram, n (%)	31,478 (34.6%)	14,542 (38.1%)	0.072	12,892 (32.9%)	14,581 (38.1%)	0.108	37,922 (34.4%)	30,266 (34.0%)	0.007
CT scan, n (%)	42,098 (46.3%)	19,281 (50.5%)	0.084	17,012 (43.5%)	19,335 (50.5%)	0.142	50,504 (45.7%)	38,860 (43.7%)	0.042
Bronchoscopy or biopsy, n (%)	2,114 (2.3%)	1,055 (2.8%)	0.028	835 (2.1%)	1,055 (2.8%)	0.040	2,925 (2.6%)	2,560 (2.9%)	0.014
Mammography, n (%)	18,732 (20.6%)	8,098 (21.2%)	0.015	8,038 (20.5%)	8,115 (21.2%)	0.017	22,892 (20.7%)	17,712 (19.9%)	0.021
Colonoscopy, n (%)	623 (0.7%)	274 (0.7%)	0.004	350 (0.9%)	271 (0.7%)	0.021	920 (0.8%)	1,132 (1.3%)	0.043
Bone-mineral density scan, n (%)	8,114 (8.9%)	3,715 (9.7%)	0.028	3,346 (8.5%)	3,726 (9.7%)	0.041	9,881 (9.0%)	7,467 (8.4%)	0.020
Influenza vaccination, n (%)	51,550 (56.7%)	22,286 (58.4%)	0.034	21,563 (55.1%)	22,333 (58.3%)	0.066	59,151 (53.6%)	40,910 (46.0%)	0.152
Non-pulmonary medications, n (%)									
Statins	54,301 (59.7%)	23,086 (60.5%)	0.015	21,776 (55.6%)	23,192 (60.6%)	0.101	63,522 (57.5%)	45,551 (51.2%)	0.127
Beta-blockers	39,344 (43.3%)	17,395 (45.6%)	0.046	16,204 (41.4%)	17,469 (45.6%)	0.086	46,189 (41.8%)	35,041 (39.4%)	0.050
Angiotensin-converting enzyme inhibitors	27,308 (30.0%)	10,880 (28.5%)	0.034	12,807 (32.7%)	10,890 (28.5%)	0.093	34,131 (30.9%)	30,951 (34.8%)	0.083
Angiotensin receptor blockers	23,134 (25.5%)	9,985 (26.2%)	0.016	8,477 (21.7%)	10,061 (26.3%)	0.109	26,374 (23.9%)	16,596 (18.7%)	0.128
Calcium channel blockers	28,891 (31.8%)	12,444 (32.6%)	0.017	11,858 (30.3%)	12,514 (32.7%)	0.052	34,174 (31.0%)	25,998 (29.2%)	0.038
Thiazide diuretics	21,826 (24.0%)	8,944 (23.4%)	0.014	9,381 (24.0%)	8,951 (23.4%)	0.014	27,063 (24.5%)	22,499 (25.3%)	0.018
Loop diuretics	25,745 (28.3%)	11,127 (29.2%)	0.018	10,909 (27.9%)	11,154 (29.1%)	0.028	30,421 (27.6%)	24,773 (27.8%)	0.007
Proton-pump inhibitors	37,162 (40.9%)	15,506 (40.6%)	0.005	15,309 (39.1%)	15,556 (40.6%)	0.031	43,681 (39.6%)	32,165 (36.2%)	0.070
H2-receptor blockers	8,368 (9.2%)	3,565 (9.3%)	0.005	3,175 (8.1%)	3,578 (9.3%)	0.044	9,485 (8.6%)	6,186 (7.0%)	0.061
Metformin	16,366 (18.0%)	6,382 (16.7%)	0.034	6,502 (16.6%)	6,394 (16.7%)	0.003	18,817 (17.0%)	12,705 (14.3%)	0.076
Sulfonylureas	7,462 (8.2%)	2,899 (7.6%)	0.023	3,252 (8.3%)	2,918 (7.6%)	0.025	9,229 (8.4%)	8,006 (9.0%)	0.023
Sodium-glucose cotransporter-2 inhibitors	2,360 (2.6%)	904 (2.4%)	0.015	401 (1.0%)	915 (2.4%)	0.106	2,331 (2.1%)	379 (0.4%)	0.151
Dipeptidyl peptidase IV inhibitors	3,553 (3.9%)	1,568 (4.1%)	0.010	1,536 (3.9%)	1,553 (4.1%)	0.007	4,012 (3.6%)	2,523 (2.8%)	0.045
Glucagon-like peptide-1 agonists	3,117 (3.4%)	1,214 (3.2%)	0.014	693 (1.8%)	1,222 (3.2%)	0.092	3,221 (2.9%)	955 (1.1%)	0.132
Benzodiazepines	22,626 (24.9%)	9,135 (23.9%)	0.022	10,165 (26.0%)	9,175 (24.0%)	0.046	25,388 (23.0%)	17,916 (20.1%)	0.070

© 2026 Feldman WB et al. *JAMA Network Open*.

SSRIs/SNRIs	31,384 (34.5%)	13,209 (34.6%)	0.002	12,936 (33.0%)	13,274 (34.7%)	0.035	36,573 (33.1%)	27,207 (30.6%)	0.055
Socioeconomic covariates[b]									
Mean copayment on other drugs, $ (SD)	19.51 (27.70)	20.77 (33.49)	0.041	20.84 (28.17)	20.81 (33.61)	0.001	21.16 (27.91)	25.17 (28.23)	0.143
Total copayment on other drugs, $ (SD)	532.54 (825.94)	558.62 (929.85)	0.030	538.90 (757.33)	559.95 (934.17)	0.025	575.56 (845.97)	660.64 (871.66)	0.099
Ratio of unique brand-to-generic drugs	0.18 (0.24)	0.19 (0.24)	0.028	0.21 (0.28)	0.19 (0.24)	0.083	0.24 (0.36)	0.39 (0.51)	0.321

Abs St Diff: absolute standardize difference; COPD: chronic obstructive pulmonary disease; SABA: short-acting beta agonist; SAMA: short-acting muscarinic antagonist; CPAP: Continuous pressure airway pressure; BiPAP: Bi-level positive airway pressure; LAMA: long-acting muscarinic antagonist; LABA: long-acting beta-agonist; ICS: inhaled corticosteroid; SSRI: selective serotonin reuptake inhibitor; SNRI: serotonin and norepinephrine reuptake inhibitor.

a. This covariate was measured using all available data for each patient (see eFigure 1).

b. These covariates exclude out-of-pocket costs on the day of cohort entry and thus would not reflect differences in costs for the index prescription.

© 2026 Feldman WB et al. *JAMA Network Open*.

eTable 3. COPD Exacerbations, Pneumonia Hospitalizations, and All-Cause Mortality in Patients Receiving Single-Inhaler Dual Therapy in the Unmatched Cohort

	Referent events	Exposure events	Referent events/ 1,000 person-years	Exposure events/ 1,000 person-years	HR (95% CI)
**Fluticasone furoate-vilanterol (exposure, n=38,164) vs. budesonide-formoterol (referent, n=90,883)**					
Moderate or severe COPD exacerbation	17,855	7,233	641.4	552.9	0.88 (0.86 to 0.91)
Moderate COPD exacerbation	16,076	6,526	571.8	494.3	0.88 (0.86 to 0.91)
Severe COPD exacerbation	2,335	851	73.1	57.1	0.80 (0.74 to 0.87)
Pneumonia hospitalization	3,579	1,662	113.3	113.0	1.03 (0.97 to 1.09)
All-cause mortality	2,837	1,321	87.8	87.7	1.01 (0.95 to 1.08)
**Fluticasone furoate-vilanterol (exposure, n=38,277) vs. fluticasone propionate-salmeterol (referent, n=39,152)**					
Moderate or severe COPD exacerbation	6,822	7,283	603.6	555.4	0.95 (0.92 to 0.98)
Moderate COPD exacerbation	5,920	6,567	518.0	496.3	0.99 (0.95 to 1.02)
Severe COPD exacerbation	1,116	863	88.0	57.7	0.69 (0.63 to 0.76)
Pneumonia hospitalization	1,593	1,660	127.2	112.5	0.93 (0.87 to 0.99)
All-cause mortality	1,175	1,317	91.5	87.2	0.98 (0.90 to 1.06)
**Fluticasone propionate-salmeterol (exposure, n=88,958 vs. budesonide-formoterol (referent, n=110,396)**					
Moderate or severe COPD exacerbation	20,904	14,139	625.7	541.7	0.86 (0.84 to 0.88)
Moderate COPD exacerbation	18,702	12,110	554.0	458.8	0.83 (0.81 to 0.84)
Severe COPD exacerbation	2,835	2,507	74.3	86.5	1.15 (1.09 to 1.21)
Pneumonia hospitalization	4,240	3,522	112.3	122.9	1.08 (1.03 to 1.13)
All-cause mortality	3,245	2,813	84.1	95.8	1.14 (1.08 to 1.19)

HR: Hazard ratio; CI: confidence interval; COPD: chronic obstructive pulmonary disease

a. This table reports outcomes in the unmatched cohort. Table 2 reports outcomes in the 1:1 propensity score-matched cohort.

© 2026 Feldman WB et al. *JAMA Network Open*.

eTable 4. Reasons for Censoring in the Analysis of First Moderate or Severe COPD Exacerbation

	Cohort 1		Cohort 2		Cohort 3	
	**Fluticasone furoate-vilanterol vs. budesonide-formoterol (n=38,070 matched pairs), n (%)[a]**		**Fluticasone furoate-vilanterol vs. fluticasone propionate-salmeterol (n=20,471 matched pairs), n (%)[b]**		**Fluticasone propionate-salmeterol vs. budesonide-formoterol (n=55,627 matched pairs), n (%)[c]**	
**Censoring reason**	Budesonide-formoterol	Fluticasone furoate-vilanterol	Fluticasone propionate-salmeterol	Fluticasone furoate-vilanterol	Budesonide-formoterol	Fluticasone propionate-salmeterol
Outcome	7,144 (18.8)	7,065 (18.6)	3,547 (17.3)	3,836 (18.7)	9,603 (17.3)	9,068 (16.3)
Death	1,066 (2.8)	1,057 (2.8)	485 (2.4)	505 (2.5)	1,309 (2.4)	1,311 (2.4)
Start of exposure different from the index exposure	169 (0.4)	345 (0.9)	114 (0.6)	91 (0.4)	438 (0.8)	515 (0.9)
End of index exposure	18,647 (49.0)	17,108 (44.9)	9,957 (48.6)	9,151 (44.7)	27,981 (50.3)	27,707 (49.8)
Maximum follow-up time	1,997 (5.2)	3,092 (8.1)	690 (3.4)	1,658 (8.1)	2,824 (5.1)	2,382 (4.3)
End of patient enrollment	1,856 (4.9)	2,142 (5.6)	1,025 (5.0)	1,131 (5.5)	2,944 (5.3)	2,978 (5.4)
Another maintenance inhaler therapy begun	7,191 (18.9)	7,261 (19.1)	4,653 (22.7)	4,099 (20.0)	10,528 (18.9)	11,666 (21.0)

a. Mean follow-up time was 112.9 days (standard deviation [SD] 90.9 days) and median follow-up 88 days (interquartile range [IQR] 54-148 days) in the group of patients receiving budesonide-formoterol, while mean follow-up was 125.1 days (SD 101.8 days) and median follow-up 88 days (IQR 58-156 days) in the group of patients receiving fluticasone furoate-vilanterol.
b. Mean follow-up time was 103.6 days (SD 82.6 days) and median follow-up 88 days (IQR 49-135 days) in the group of patients receiving fluticasone propionate-salmeterol, while mean follow-up time was 123.1 days (SD 101.9 days) and median follow-up 88 days (IQR 55-151 days) in the group of patients receiving fluticasone furoate-vilanterol.
c. Mean follow-up time was 109.1 days (SD 89.2 days) and median follow-up 88 days (IQR 53-136 days) in the group of patients receiving budesonide-formoterol, while mean follow-up time was 105.4 days (SD 85.9 days) and 88 days (IQR 49-132 days) in the group of patients receiving fluticasone propionate-salmeterol.
d. This includes a long-acting muscarinic antagonist (LAMA), long-acting beta agonist (LABA), inhaled corticosteroid (ICS), LAMA-LABA, ICS-LAMA-LABA, or an ICS-LABA other than the reference or exposure therapy.

© 2026 Feldman WB et al. *JAMA Network Open*.

eTable 5. Reasons for Censoring in the Analysis of First Pneumonia Hospitalization

	Cohort 1		Cohort 2		Cohort 3	
	**Fluticasone furoate-vilanterol vs. budesonide-formoterol (n=38,070 matched pairs), n (%)[a]**		**Fluticasone furoate-vilanterol vs. fluticasone propionate-salmeterol (n=20,471 matched pairs), n (%)[b]**		**Fluticasone propionate-salmeterol vs. budesonide-formoterol (n=55,627 matched pairs), n (%)[c]**	
**Censoring reason**	Budesonide-formoterol	Fluticasone furoate-vilanterol	Fluticasone propionate-salmeterol	Fluticasone furoate-vilanterol	Budesonide-formoterol	Fluticasone propionate-salmeterol
Outcome	1,493 (3.9)	1,655 (4.3)	825 (4.0)	879 (4.3)	2,179 (3.9)	2,184 (3.9)
Death	1,000 (2.6)	1,000 (2.6)	451 (2.2)	491 (2.4)	1,236 (2.2)	1,211 (2.2)
Start of exposure different from the index exposure	196 (0.5)	405 (1.1)	131 (0.6)	110 (0.5)	497 (0.9)	611 (1.1)
End of index exposure	21,987 (57.8)	19,858 (52.2)	11,551 (56.4)	10,641 (52.0)	32,414 (58.3)	31,718 (57.0)
Maximum follow-up time	2,937 (7.7)	4,301 (11.3)	1,014 (5.0)	2,342 (11.4)	4,102 (7.4)	3,363 (6.0)
End of patient enrollment	2,132 (5.6)	2,430 (6.4)	1,174 (5.7)	1,296 (6.3)	3,278 (5.9)	3,341 (6.0)
Another maintenance inhaler therapy begun	8,325 (21.9)	8,421 (22.1)	5,325 (26.0)	4,712 (23.0)	11,921 (21.4)	13,199 (23.7)

a. Mean follow-up time was 127.7 days (standard deviation [SD] 97.3 days) and median follow-up 88 days (interquartile range [IQR] 86-148 days) in the group of patients receiving budesonide-formoterol, while mean follow-up was 140.6 days (SD 108.2 days) and median follow-up 88 days (IQR 86-185 days) in the group of patients receiving fluticasone furoate-vilanterol.
b. Mean follow-up time was 115.3 days (SD 88.2 days) and median follow-up 88 days (IQR 70-148 days) in the group of patients receiving fluticasone propionate-salmeterol, while mean follow-up time was 138.9 days (SD 109.0 days) and median follow-up 88 days (IQR 82-183 days) in the group of patients receiving fluticasone furoate-vilanterol.
c. Mean follow-up time was 122.0 days (SD 96.0 days) and median follow-up 88 days (IQR 78-148 days) in the group of patients receiving budesonide-formoterol, while mean follow-up time was 116.7 days (SD 91.8 days) and 88 days (IQR 71-148 days) in the group of patients receiving fluticasone propionate-salmeterol.
d. This includes a long-acting muscarinic antagonist (LAMA), long-acting beta agonist (LABA), inhaled corticosteroid (ICS), LAMA-LABA, ICS-LAMA-LABA, or an ICS-LABA other than the reference or exposure therapy.

© 2026 Feldman WB et al. *JAMA Network Open*.

eFigure 1. Study Design Comparing New Users of ICS-LABAs Included in the Analysis

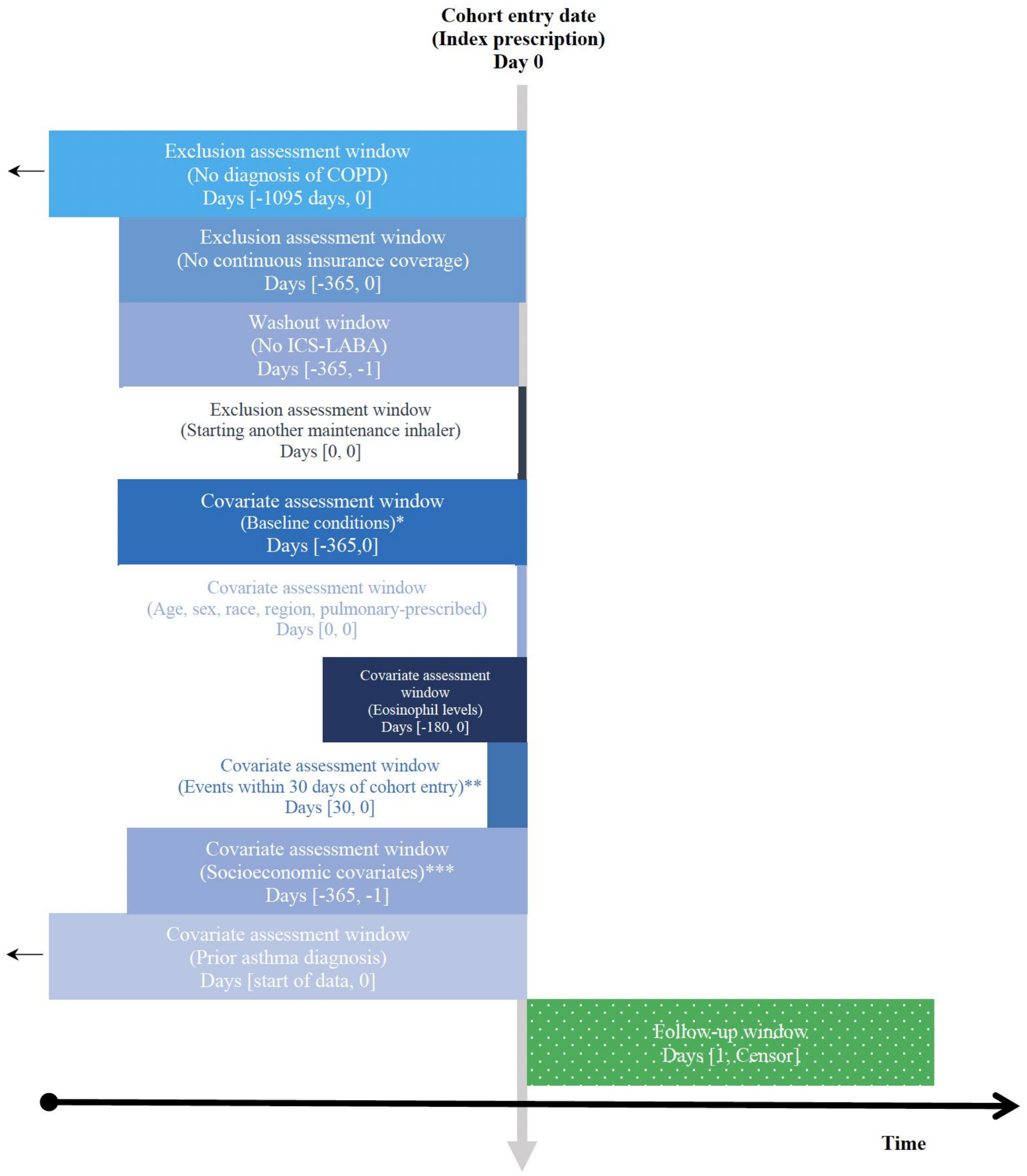

This graphical representation of study design shows how exclusion criteria were applied prior to cohort entry and how covariates were assessed.[5] The same approach was used when analyzing all 3 cohorts: (1) fluticasone furoate-vilanterol vs. budesonide-formoterol; (2) fluticasone furoate-vilanterol vs. fluticasone propionate-salmeterol; and (3) budesonide-formoterol vs. fluticasone propionate-salmeterol.

*This includes all covariates except eosinophil levels, events within 30 days and asthma diagnosis codes.

**Events within 30 days include COPD exacerbations and fills of respiratory antibiotics.

***This category includes mean copayments, total copayments, and ratio of brand to generic fills.

© 2026 Feldman WB et al. *JAMA Network Open*.

eFigure 2. Cohort Composition

A. Fluticasone furoate-vilanterol vs. budesonide-formoterol

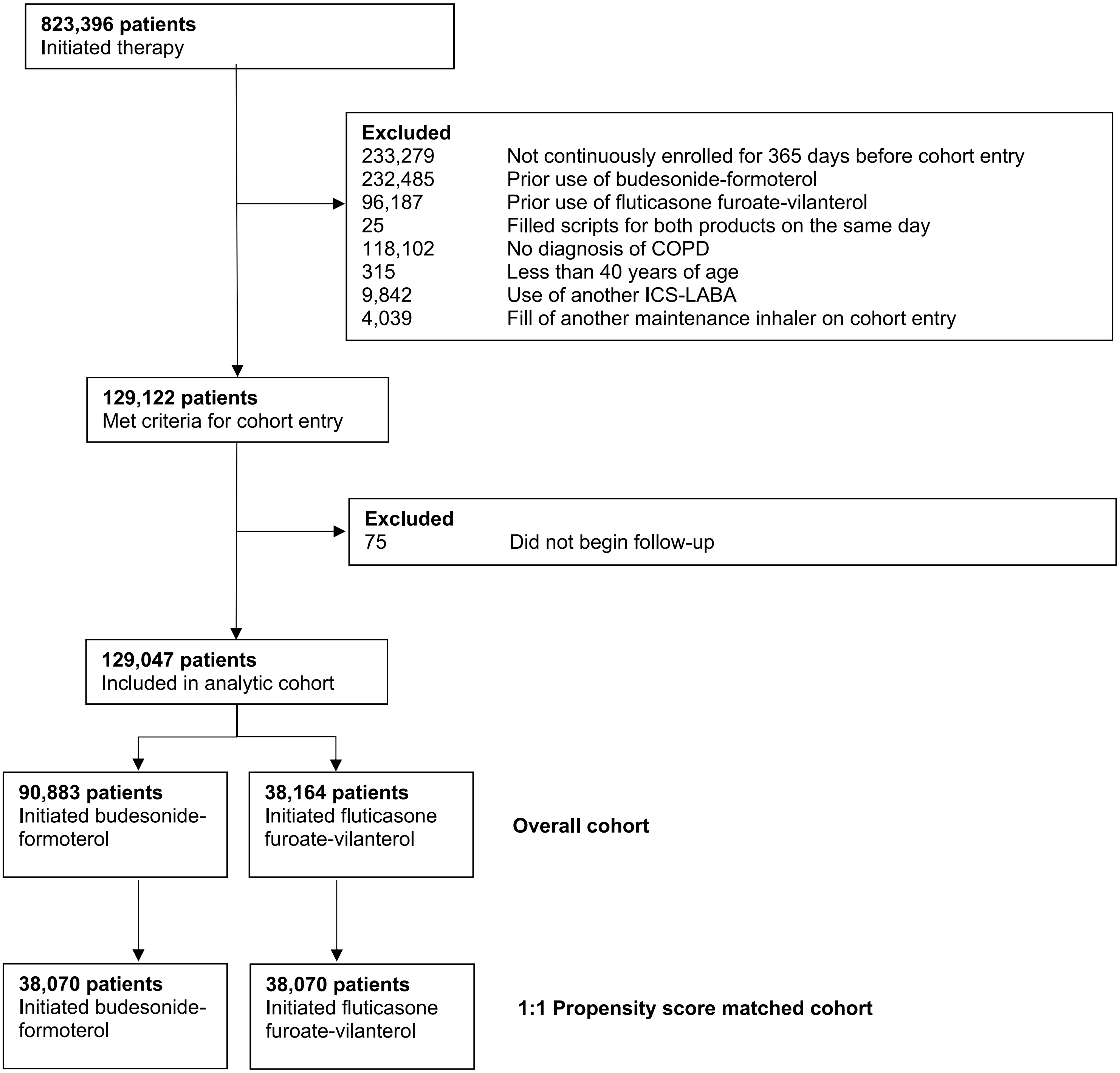

© 2026 Feldman WB et al. *JAMA Network Open*.

B. Fluticasone furoate-vilanterol vs. fluticasone propionate-salmeterol

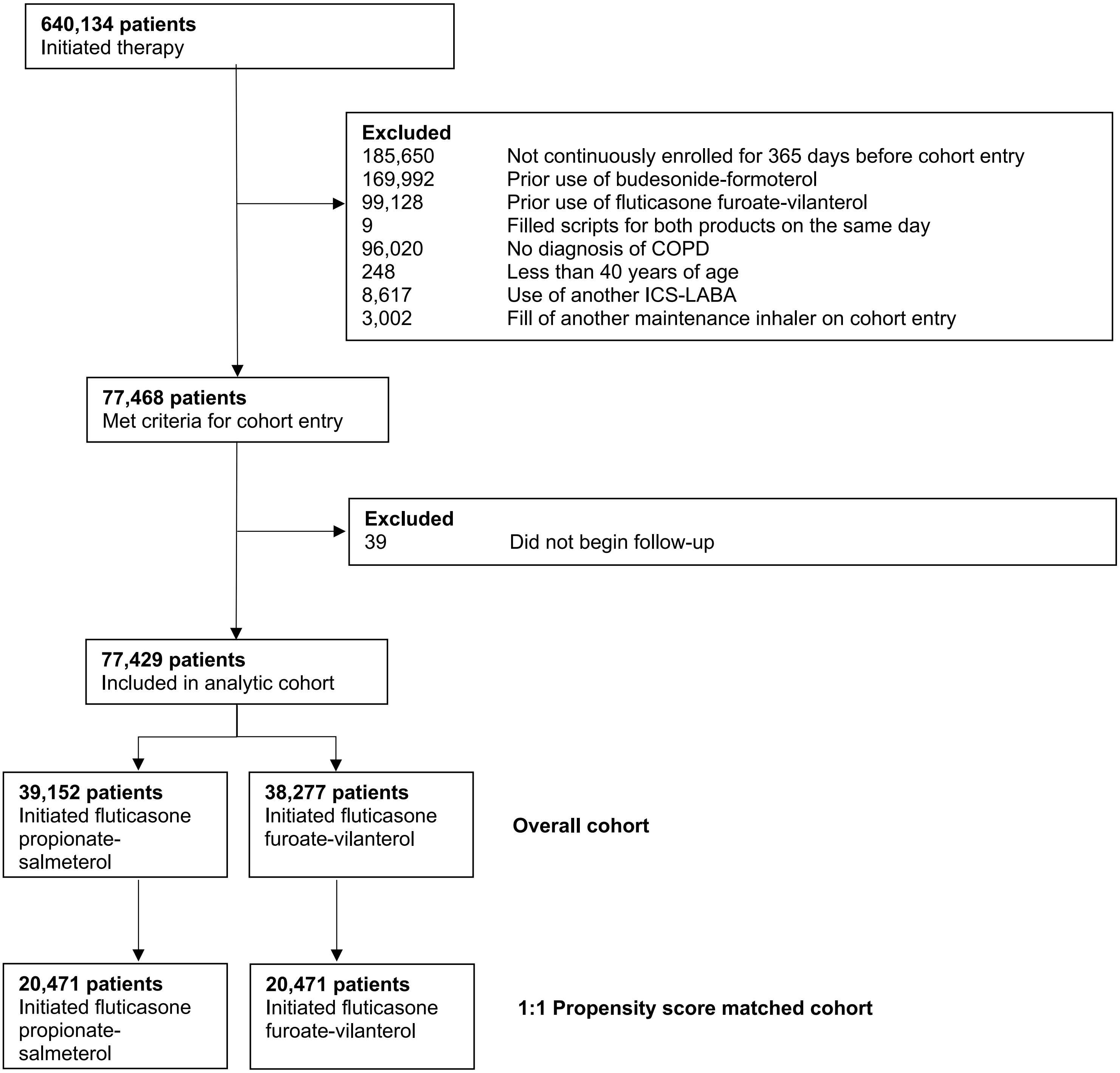

© 2026 Feldman WB et al. *JAMA Network Open*.

C. Fluticasone propionate-salmeterol vs. budesonide-formoterol

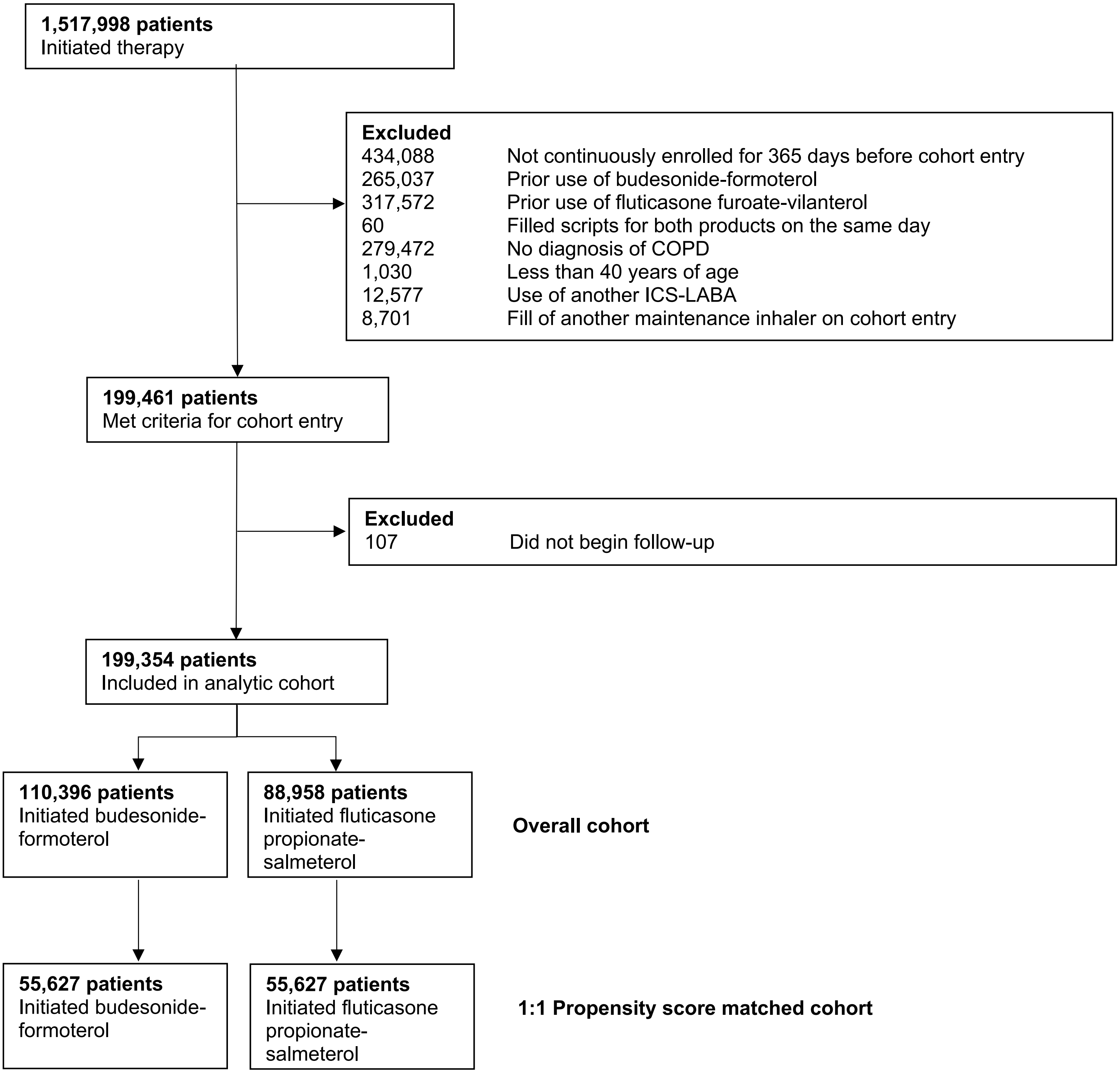

© 2026 Feldman WB et al. *JAMA Network Open*.

eFigure 3. Propensity Score Distributions Before and After Matching

A: Fluticasone furoate-vilanterol vs. budesonide-formoterol

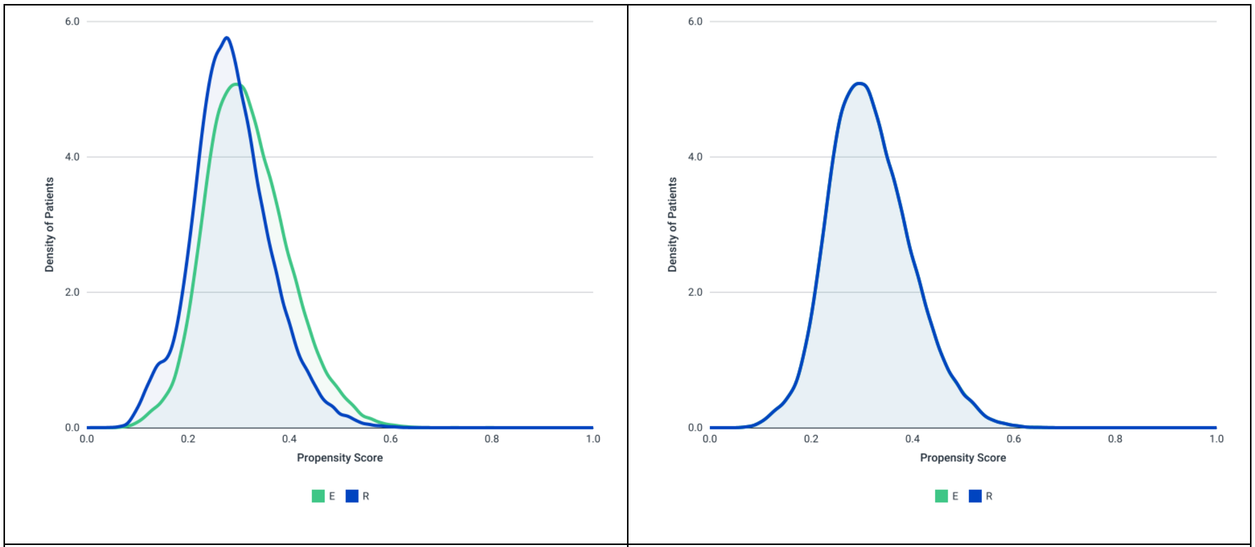

Propensity score distributions before matching	Propensity score distributions after matching

E: Exposure (fluticasone furoate-vilanterol); R: Referent (budesonide-formoterol)

B. Fluticasone furoate-vilanterol vs. fluticasone propionate-salmeterol

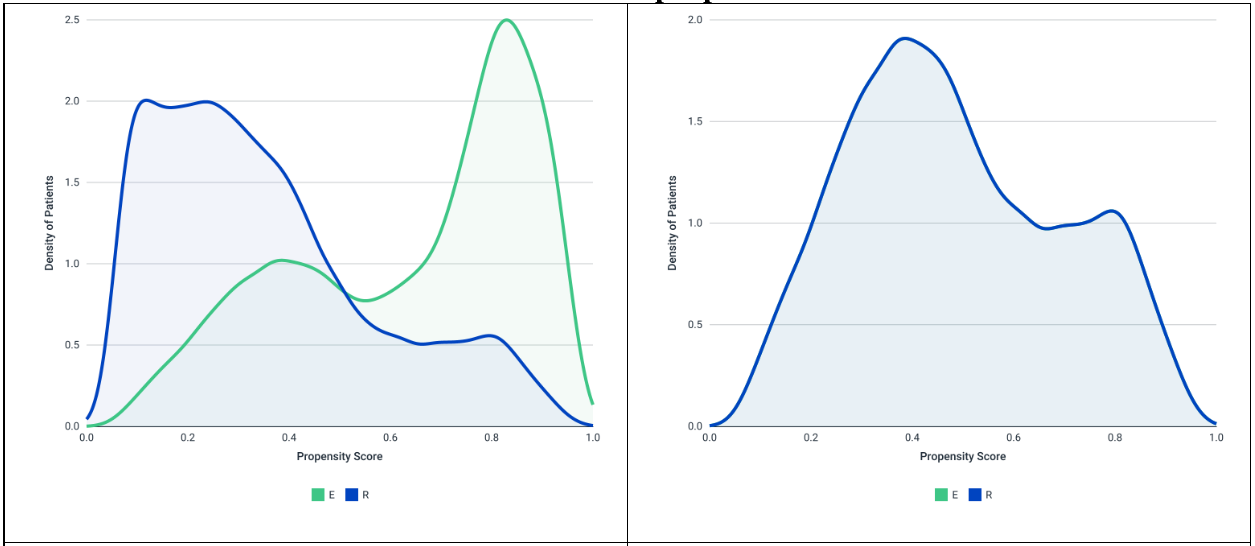

Propensity score distributions before matching	Propensity score distributions after matching

E: Exposure (fluticasone furoate-vilanterol); R: Referent (fluticasone propionate-salmeterol)

© 2026 Feldman WB et al. *JAMA Network Open*.

C. Fluticasone propionate-salmeterol vs. budesonide-formoterol

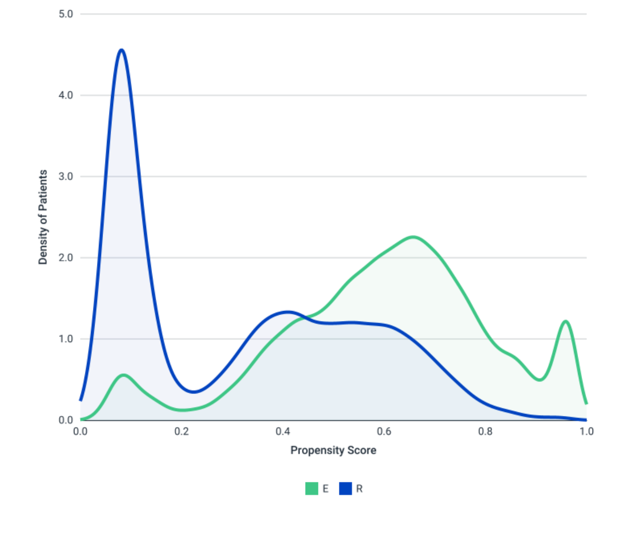

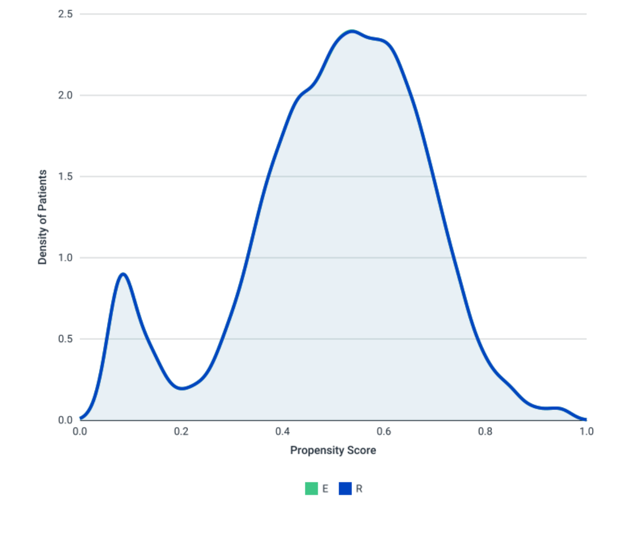

Propensity score distributions before matching	Propensity score distributions after matching

E: Exposure (fluticasone propionate-salmeterol); R: Referent (budesonide-formoterol)

© 2026 Feldman WB et al. *JAMA Network Open*.

eFigure 4. Kaplan-Meier Plots for First Moderate or Severe COPD Exacerbation

A: Fluticasone furoate-vilanterol vs. budesonide-formoterol

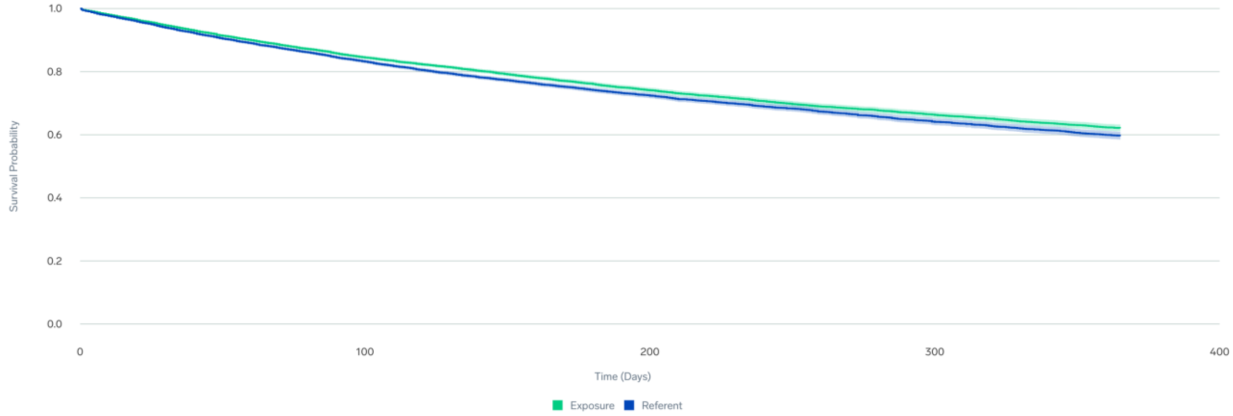

Exposure: fluticasone furoate-vilanterol; Referent: budesonide-formoterol

B. Fluticasone furoate-vilanterol vs. fluticasone propionate-salmeterol

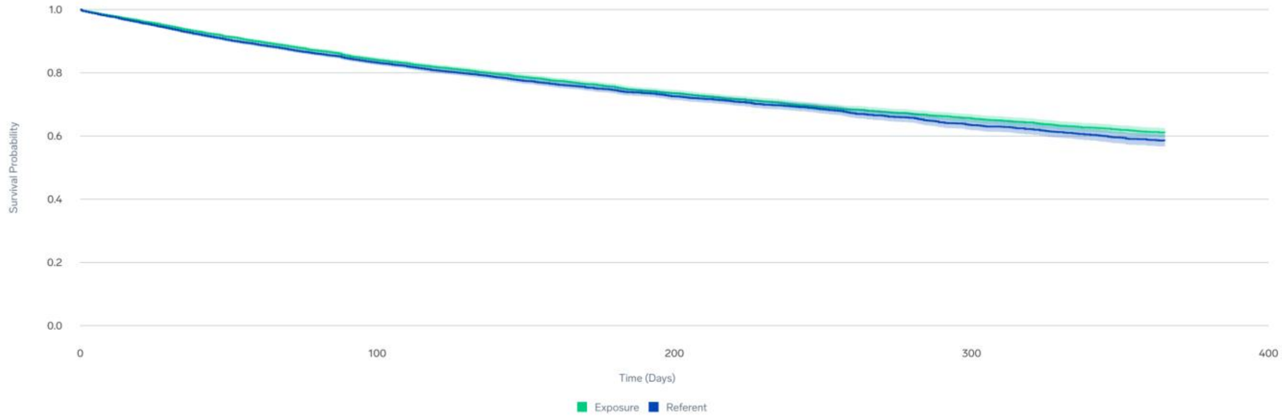

Exposure: fluticasone furoate-vilanterol; Referent: fluticasone propionate-salmeterol

C. Fluticasone propionate-salmeterol vs. budesonide-formoterol

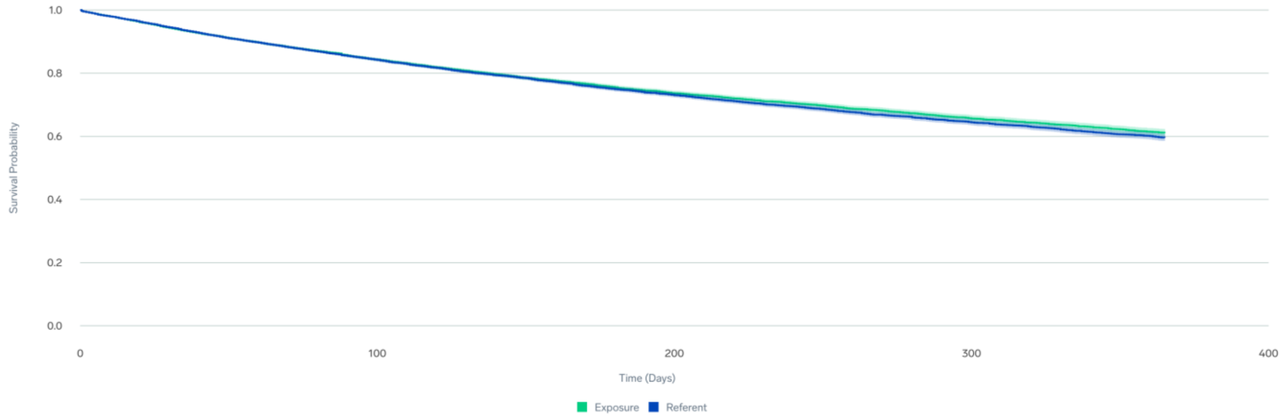

Exposure: fluticasone propionate-salmeterol; Referent: budesonide-formoterol

© 2026 Feldman WB et al. *JAMA Network Open*.

This figure shows the probability of experiencing no moderate or severe COPD exacerbation in the 365 days of follow-up for patients in the 1:1 propensity-score matched cohorts: fluticasone furoate-vilanterol vs. budesonide-formoterol (Panel A); fluticasone furoate-vilanterol vs. fluticasone propionate-salmeterol (Panel B); fluticasone propionate-salmeterol vs. budesonide-formoterol (Panel C).

© 2026 Feldman WB et al. *JAMA Network Open*.

eFigure 5. Kaplan-Meier Plots for First Pneumonia Hospitalization

A: Fluticasone furoate-vilanterol vs. budesonide-formoterol

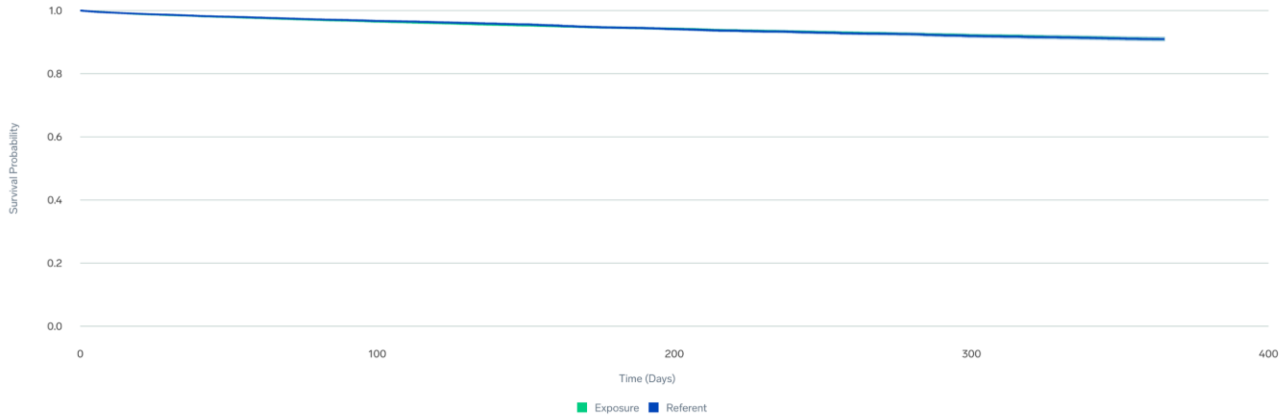

Exposure: fluticasone furoate-vilanterol; Referent: budesonide-formoterol

B. Fluticasone furoate-vilanterol vs. fluticasone propionate-salmeterol

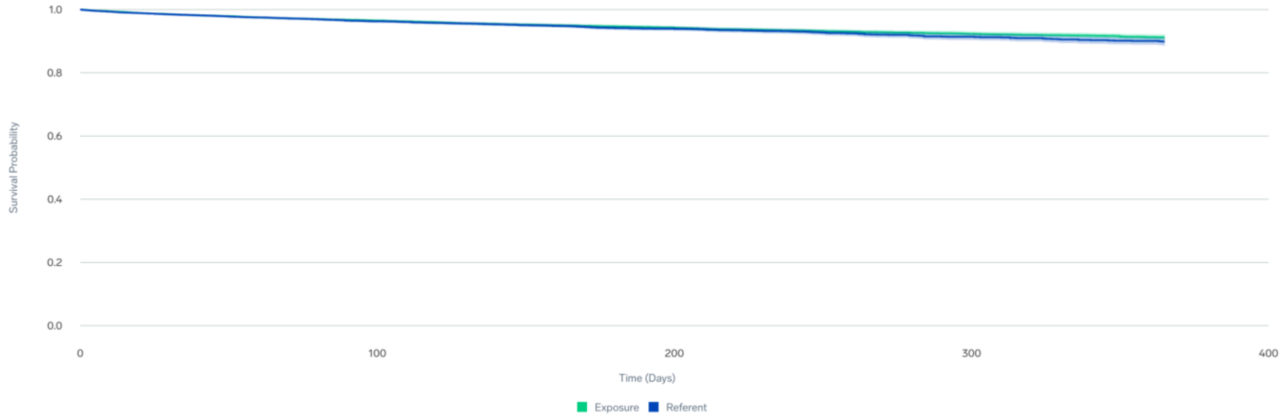

Exposure: fluticasone furoate-vilanterol; Referent: fluticasone propionate-salmeterol

C. Fluticasone propionate-salmeterol vs. budesonide-formoterol

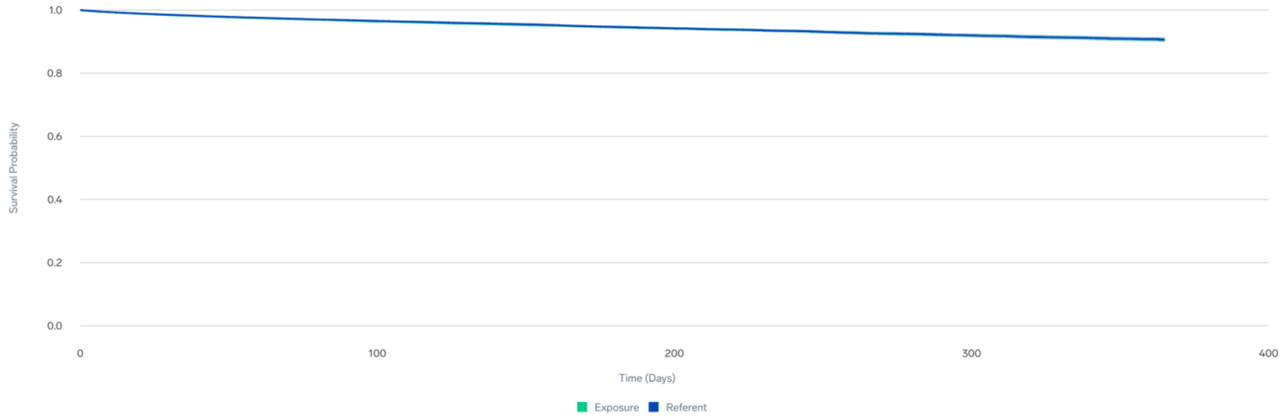

Exposure: fluticasone propionate-salmeterol; Referent: budesonide-formoterol

© 2026 Feldman WB et al. *JAMA Network Open*.

This figure shows the probability of experiencing no pneumonia hospitalization in the 365 days of follow-up for patients in the 1:1 propensity-score matched cohorts: fluticasone furoate-vilanterol vs. budesonide-formoterol (Panel A); fluticasone furoate-vilanterol vs. fluticasone propionate-salmeterol (Panel B); fluticasone propionate-salmeterol vs. budesonide-formoterol (Panel C).

© 2026 Feldman WB et al. *JAMA Network Open*.

eFigure 6. Sensitivity Analysis for First Pneumonia Hospitalization

A: Fluticasone furoate-vilanterol vs. budesonide-formoterol

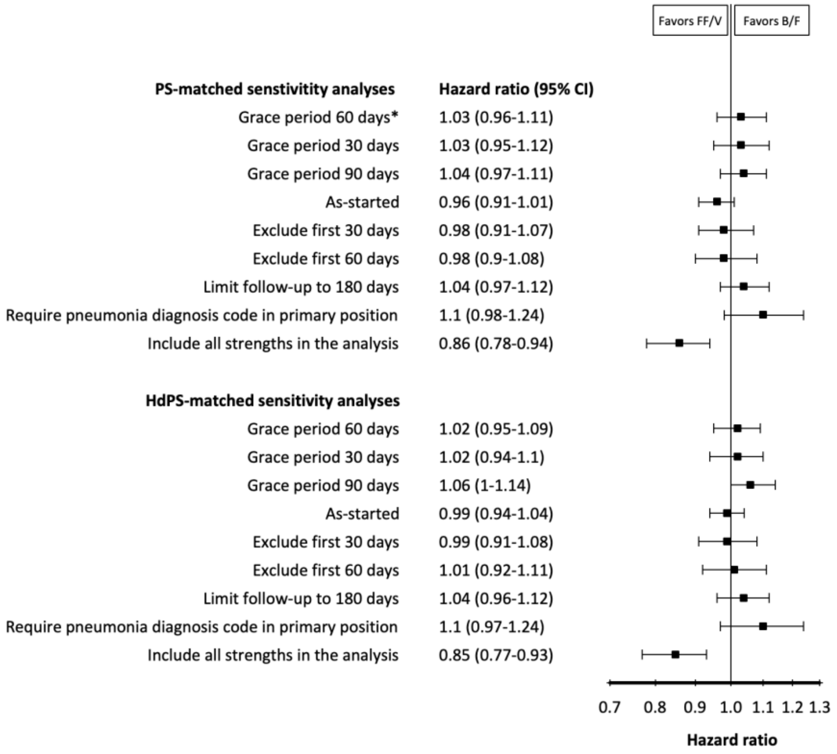

B: Fluticasone furoate-vilanterol vs. fluticasone propionate-salmeterol

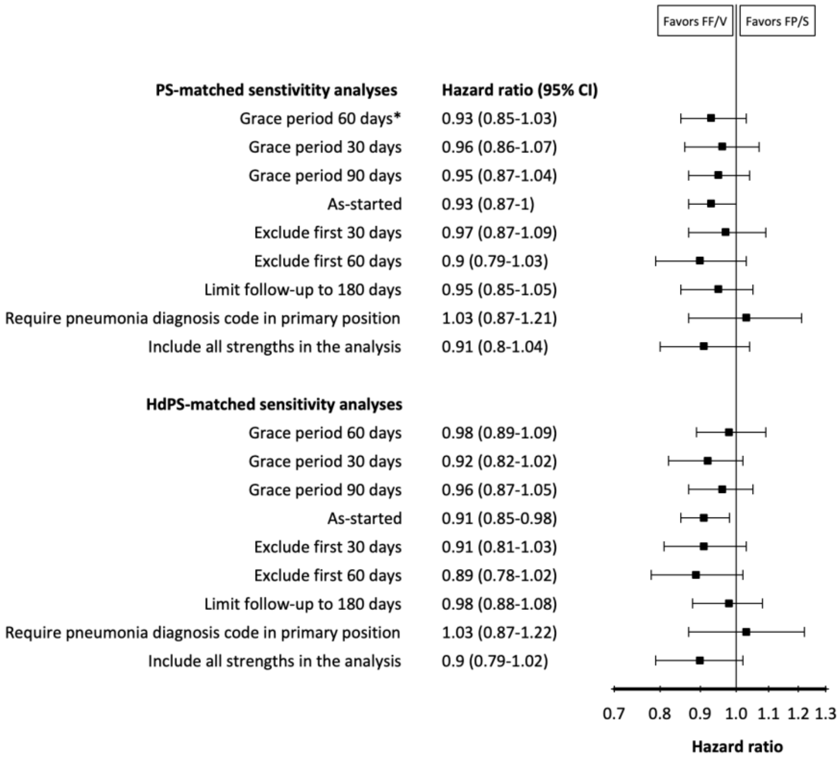

© 2026 Feldman WB et al. *JAMA Network Open*.

C: Budesonide-formoterol vs. fluticasone propionate-salmeterol

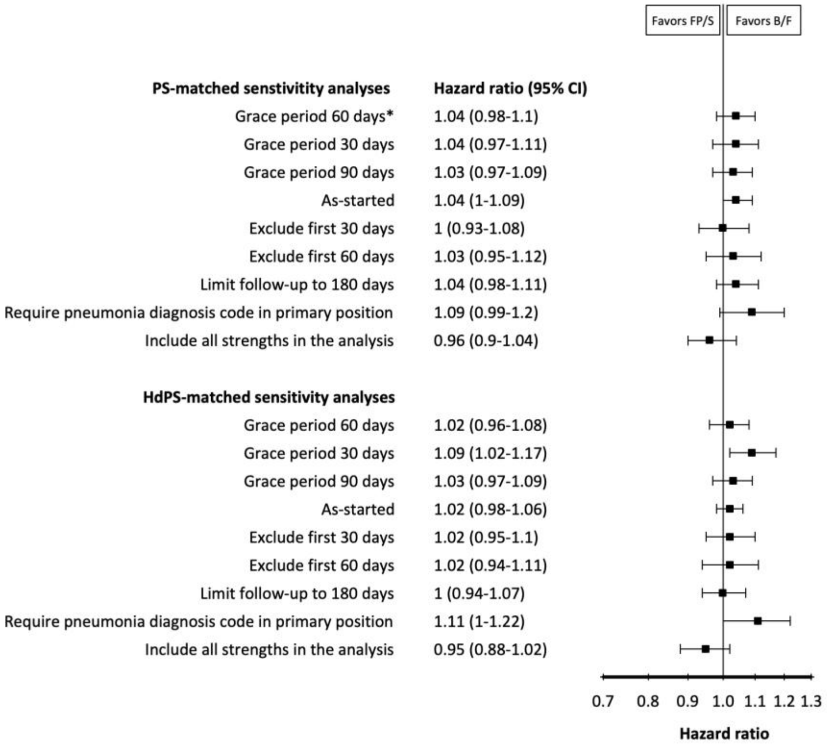

FF/V: fluticasone furoate-vilanterol; B/F: budesonide-formoterol; FP/S: fluticasone propionate-salmeterol; CI: confidence interval.
*Primary analysis.
This figure shows the hazard ratios and 95% confidence intervals of first pneumonia hospitalization in new users of the combination inhaled corticosteroids-long-acting beta agonists under investigation across a range of prespecified sensitivity analyses: fluticasone furoate-vilanterol vs. budesonide-formoterol (Panel A); fluticasone furoate-vilanterol vs. fluticasone propionate-salmeterol (Panel B); fluticasone propionate-salmeterol vs. budesonide-formoterol (Panel C).

© 2026 Feldman WB et al. *JAMA Network Open*.

eFigure 7. Sensitivity Analysis for First Moderate COPD Exacerbation

A: Fluticasone furoate-vilanterol vs. budesonide-formoterol

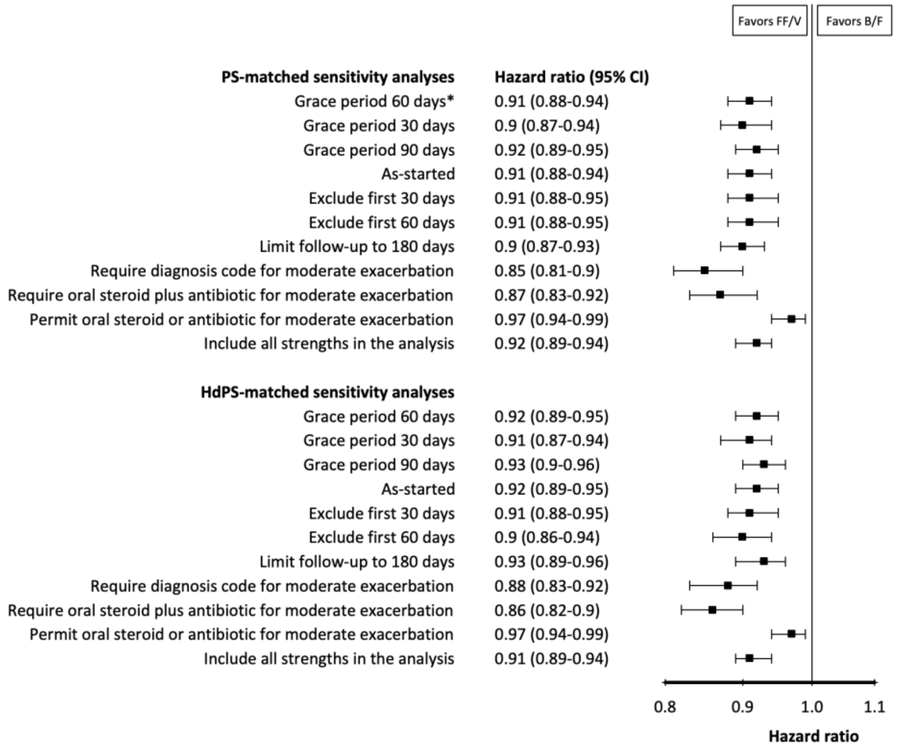

B: Fluticasone furoate-vilanterol vs. fluticasone propionate-salmeterol

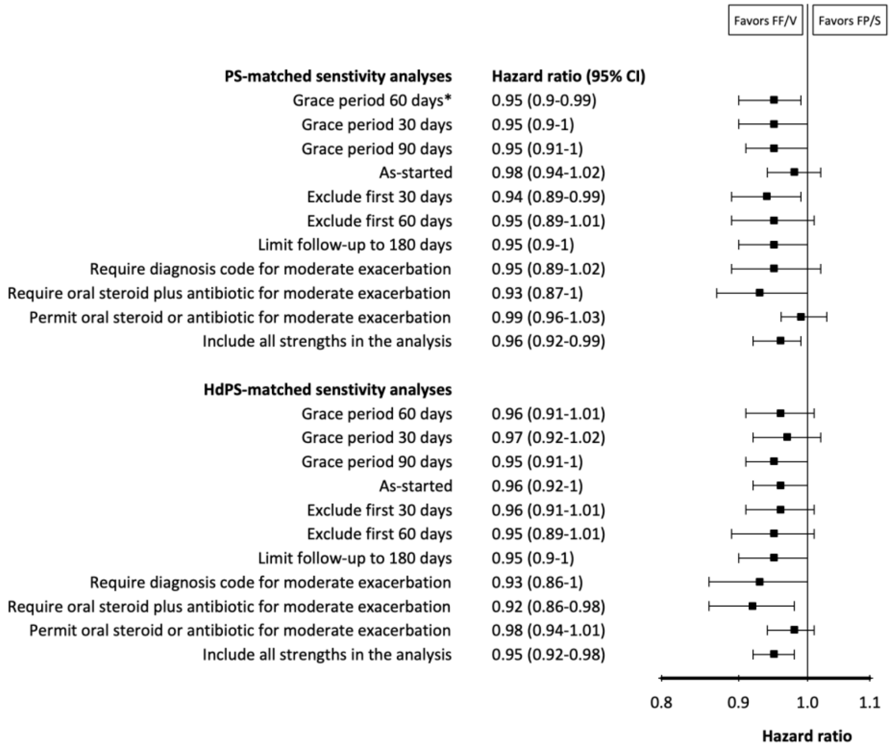

© 2026 Feldman WB et al. *JAMA Network Open*.

C: Budesonide-formoterol vs. fluticasone propionate-salmeterol

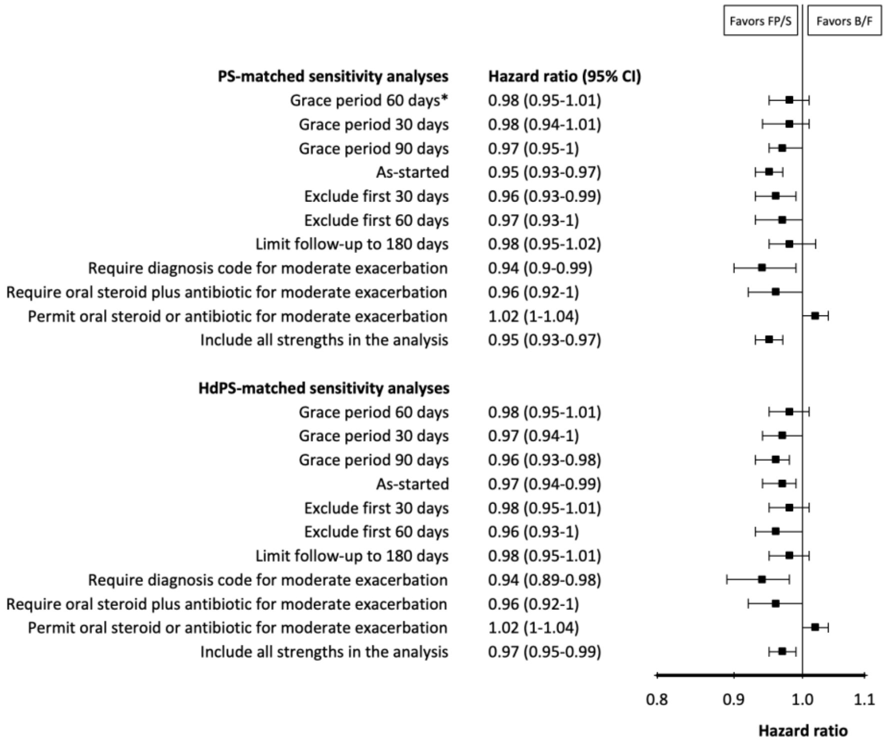

FF/V: fluticasone furoate-vilanterol; B/F: budesonide-formoterol; FP/S: fluticasone propionate-salmeterol; CI: confidence interval.

*Primary analysis.

This figure shows the hazard ratios and 95% confidence intervals of first moderate COPD exacerbation in new users of the combination inhaled corticosteroids-long-acting beta agonists under investigation across a range of prespecified sensitivity analyses: fluticasone furoate-vilanterol vs. budesonide-formoterol (Panel A); fluticasone furoate-vilanterol vs. fluticasone propionate-salmeterol (Panel B); fluticasone propionate-salmeterol vs. budesonide-formoterol (Panel C).

© 2026 Feldman WB et al. *JAMA Network Open*.

eFigure 8. Sensitivity Analysis for First Severe COPD Exacerbation

A: Fluticasone furoate-vilanterol vs. budesonide-formoterol

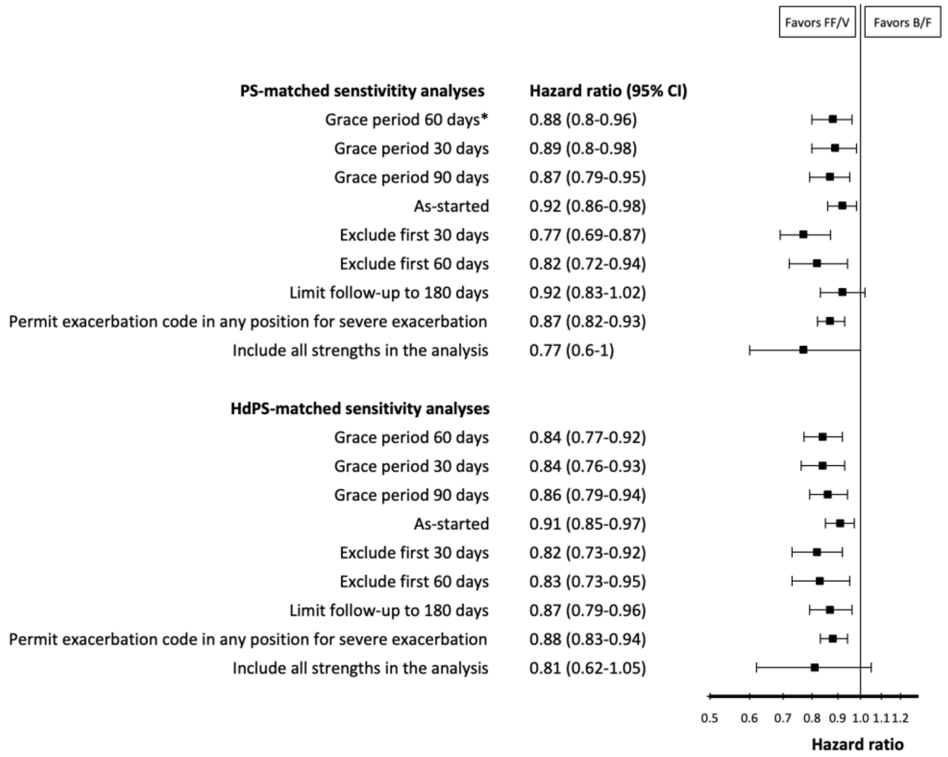

B: Fluticasone furoate-vilanterol vs. fluticasone propionate-salmeterol

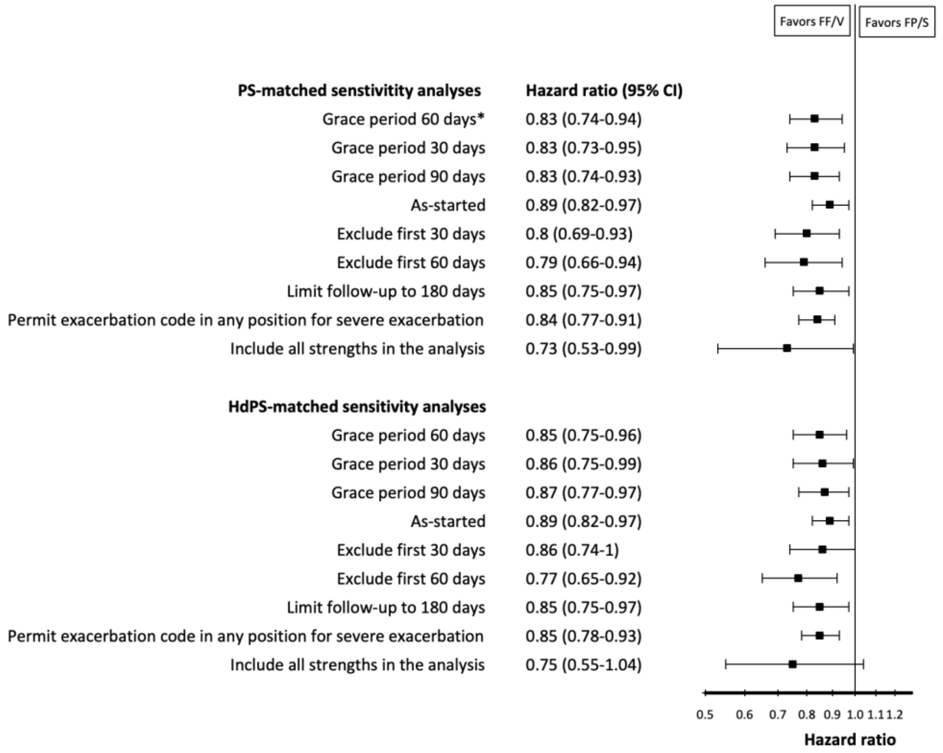

© 2026 Feldman WB et al. *JAMA Network Open*.

C: Budesonide-formoterol vs. fluticasone propionate-salmeterol

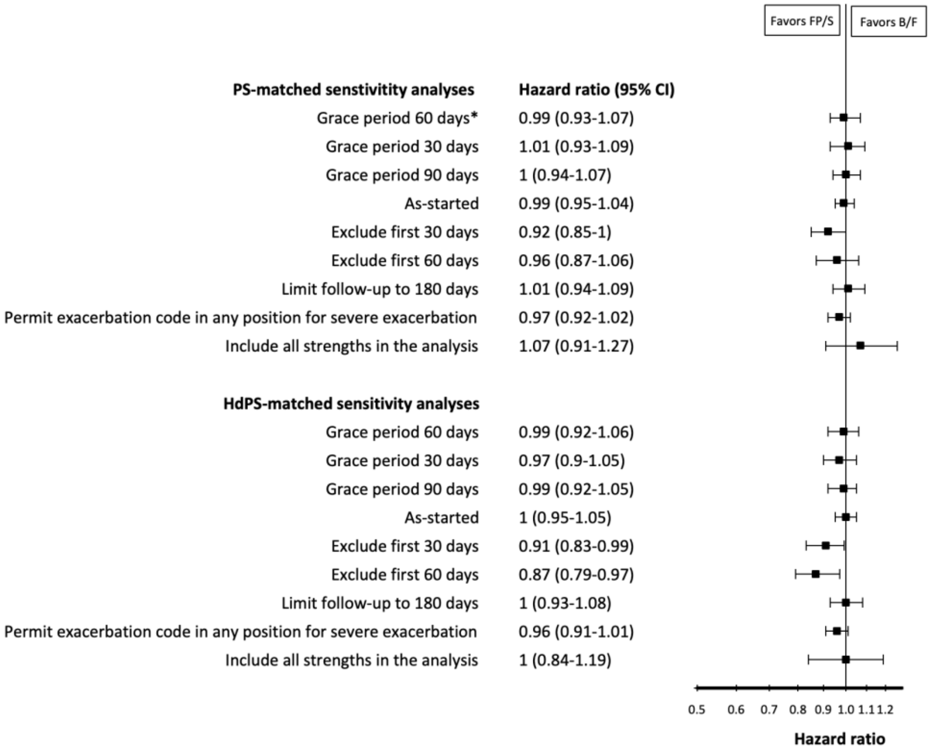

FF/V: fluticasone furoate-vilanterol; B/F: budesonide-formoterol; FP/S: fluticasone propionate-salmeterol; CI: confidence interval.

*Primary analysis.

This figure shows the hazard ratios and 95% confidence intervals of first severe COPD exacerbation in new users of the combination inhaled corticosteroids-long-acting beta agonists under investigation across a range of prespecified sensitivity analyses: fluticasone furoate-vilanterol vs. budesonide-formoterol (Panel A); fluticasone furoate-vilanterol vs. fluticasone propionate-salmeterol (Panel B); fluticasone propionate-salmeterol vs. budesonide-formoterol (Panel C).

© 2026 Feldman WB et al. *JAMA Network Open*.

eFigure 9. Sensitivity Analysis for All-Cause Mortality

A: Fluticasone furoate-vilanterol vs. budesonide-formoterol

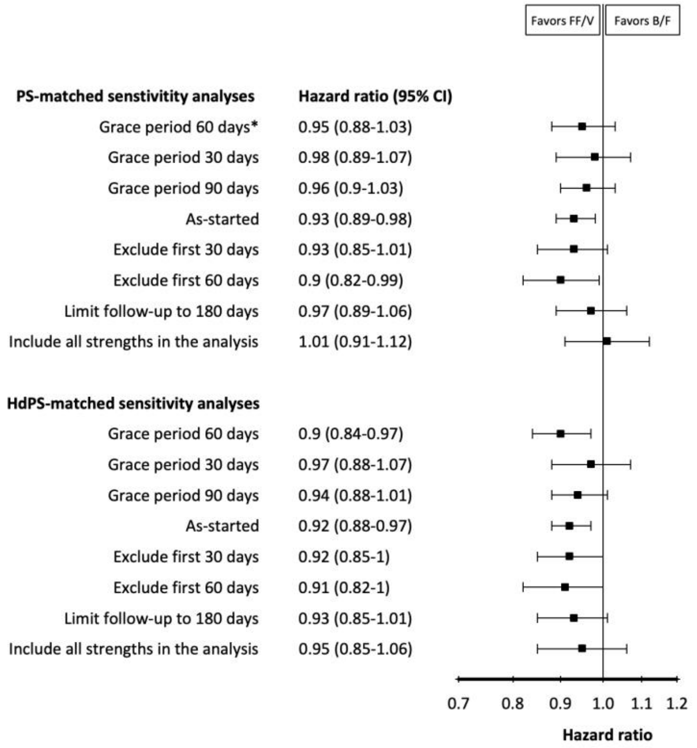

B: Fluticasone furoate-vilanterol vs. fluticasone propionate-salmeterol

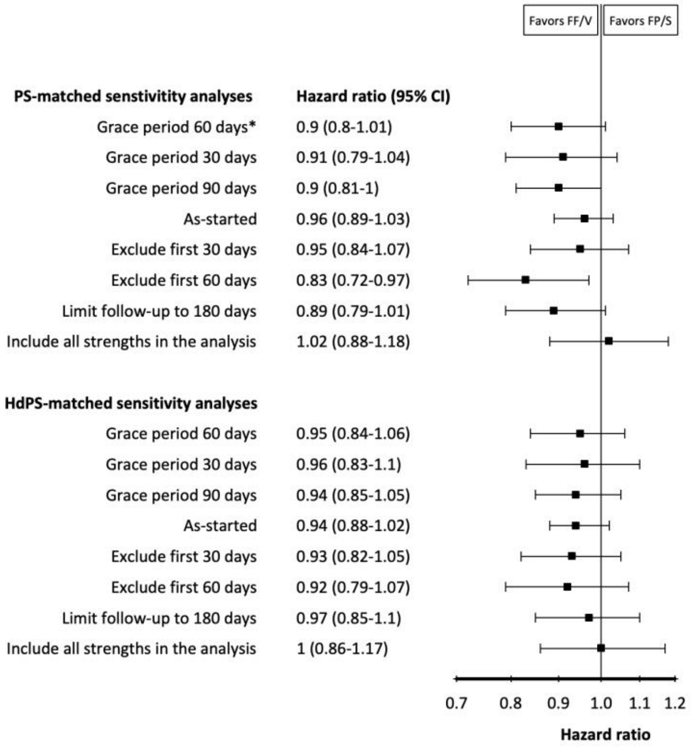

© 2026 Feldman WB et al. *JAMA Network Open*.

C: Budesonide-formoterol vs. fluticasone propionate-salmeterol

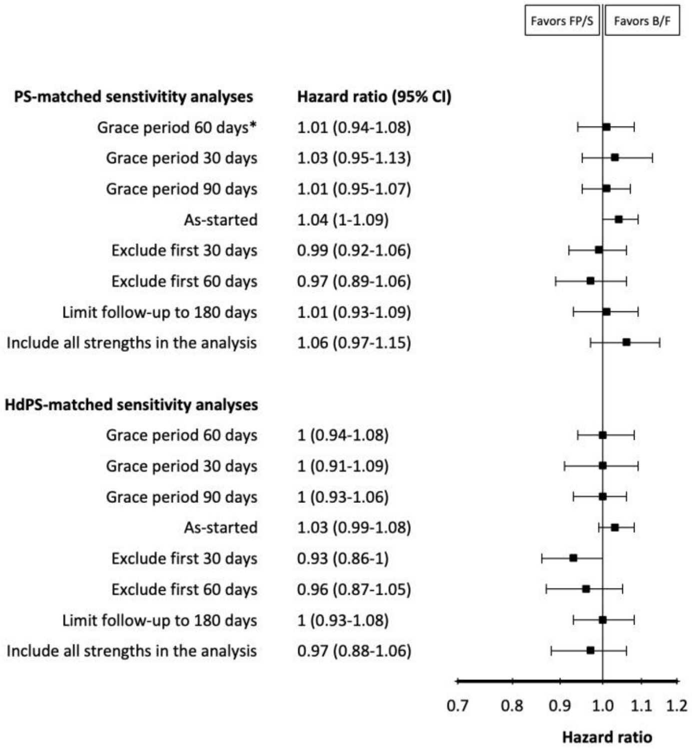

FF/V: fluticasone furoate-vilanterol; B/F: budesonide-formoterol; FP/S: fluticasone propionate-salmeterol; CI: confidence interval.
*Primary analysis.
This figure shows the hazard ratios and 95% confidence intervals of all-cause mortality in new users of the combination inhaled corticosteroids-long-acting beta agonists under investigation across a range of prespecified sensitivity analyses: fluticasone furoate-vilanterol vs. budesonide-formoterol (Panel A); fluticasone furoate-vilanterol vs. fluticasone propionate-salmeterol (Panel B); fluticasone propionate-salmeterol vs. budesonide-formoterol (Panel C).

© 2026 Feldman WB et al. *JAMA Network Open*.

eFigure 10. Subgroup Analysis for First Pneumonia Hospitalization

A: Fluticasone furoate-vilanterol vs. budesonide-formoterol

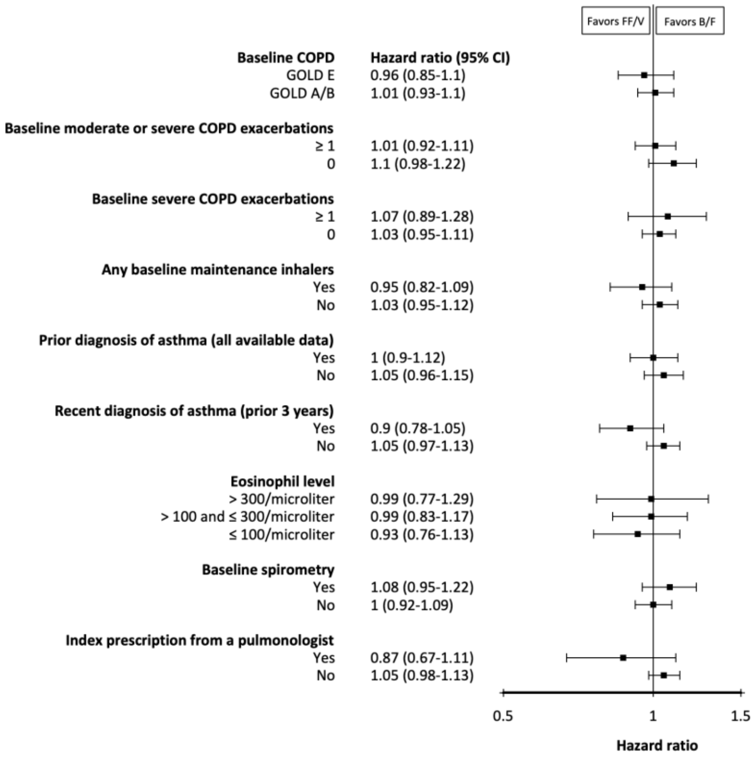

B: Fluticasone furoate-vilanterol vs. fluticasone propionate-salmeterol

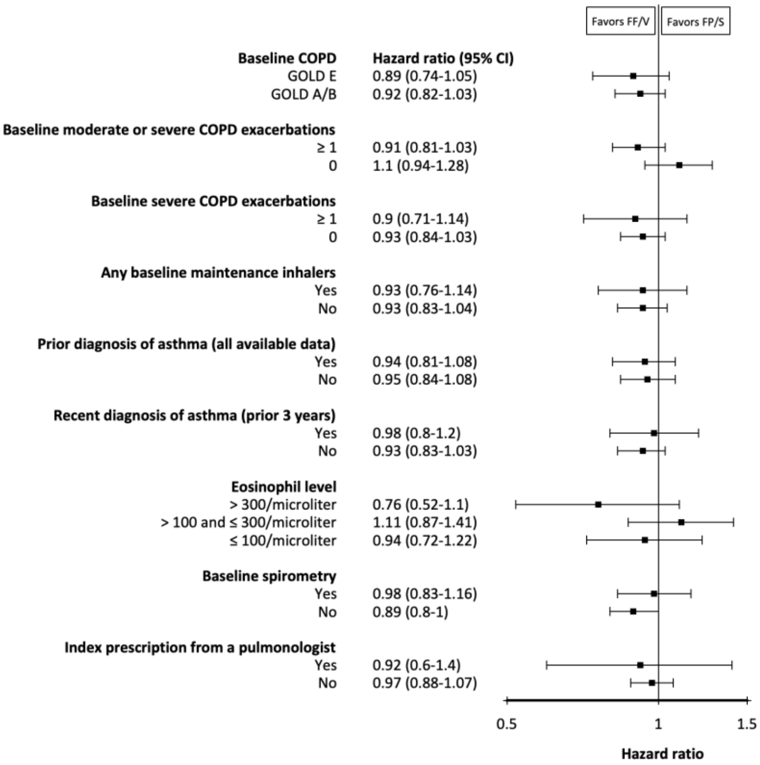

© 2026 Feldman WB et al. *JAMA Network Open*.

C: Budesonide-formoterol vs. fluticasone propionate-salmeterol

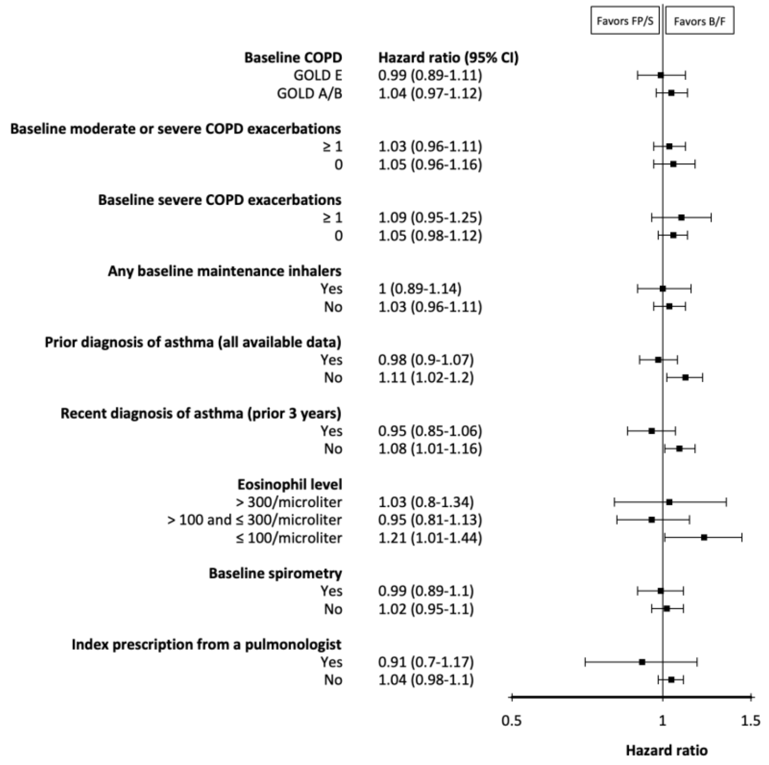

FF/V: fluticasone furoate-vilanterol; B/F: budesonide-formoterol; FP/S: fluticasone propionate-salmeterol; CI: confidence interval.

This figure shows the hazard ratios and 95% confidence intervals of first pneumonia hospitalization in new users of the combination inhaled corticosteroids-long-acting beta agonists under investigation across a range of prespecified subgroup analyses: fluticasone furoate-vilanterol vs. budesonide-formoterol (Panel A); fluticasone furoate-vilanterol vs. fluticasone propionate-salmeterol (Panel B); fluticasone propionate-salmeterol vs. budesonide-formoterol (Panel C).

© 2026 Feldman WB et al. *JAMA Network Open*.

eReferences

1. Global Iniative for Obstructive Lung Disease (GOLD). Global Strategy for Prevention, Diagnosis and Management of COPD: 2025 Report. Available online at: https://goldcopd.org/2025-gold-report/. Accessed Juky 14, 2025.
2. Gagne JJ, Glynn RJ, Avorn J, Levin R, Schneeweiss S. A combined comorbidity score predicted mortality in elderly patients better than existing scores. *J Clin Epidemiol*. 2011;64(7):749-59.
3. Kim DH, Schneeweiss S, Glynn RJ, Lipsitz LA, Rockwood K, Avorn J. Measuring Frailty in Medicare Data: Development and Validation of a Claims-Based Frailty Index. *J Gerontol A Biol Sci Med Sci*. 2018;73(7):980-987. PMC6001883.
4. Gershon AS, Wang C, Guan J, Vasilevska-Ristovska J, Cicutto L, To T. Identifying individuals with physcian diagnosed COPD in health administrative databases. *COPD*. 2009;6(5):388-94.
5. Schneeweiss S, Rassen JA, Brown JS, et al. Graphical Depiction of Longitudinal Study Designs in Health Care Databases. *Ann Intern Med*. 2019;170(6):398-406.

© 2026 Feldman WB et al. *JAMA Network Open*.
