# Supplementary material for: Fluticasone- vs Budesonide-Based Dual Therapy for COPD
Source: JAMA Netw Open. 2026 Mar 9;9(3):e260959. doi: 10.1001/jamanetworkopen.2026.0959 (PMC12973110; doi:10.1001/jamanetworkopen.2026.0959)
Supplement: Supplement 2. — Data Sharing Statement [file jamanetwopen-e260959-s002.pdf]

Data Sharing Statement

Feldman. Fluticasone- vs Budesonide-Based Dual Therapy for COPD. *JAMA Netw Open*. Published March 09, 2026. doi:10.1001/jamanetworkopen.2026.0959

Data
Data available: No

Additional Information
Explanation for why data not available: We are precluded from sharing individual patient level data by our Data Use Agreement with Optum. However, researchers interested in using this data can contact Optum directly.
